# Circular RNAs modulate the floral fate acquisition in soybean shoot apical meristem

**DOI:** 10.1186/s12870-023-04319-3

**Published:** 2023-06-16

**Authors:** Saeid Babaei, Mohan B. Singh, Prem L. Bhalla

**Affiliations:** Plant Molecular Biology and Biotechnology Laboratory, Faculty of Science, The University of Melbourne, Parkville, Melbourne, VIC 3010 Australia

**Keywords:** Soybean, *Glycine max*, Shoot apical meristem, Floral transition, CircRNA, Hormonal signalling, RNA sequencing

## Abstract

**Background:**

Soybean (*Glycine max*), a major oilseed and protein source, requires a short-day photoperiod for floral induction. Though key transcription factors controlling flowering have been identified, the role of the non-coding genome is limited. Circular RNAs (circRNAs) recently emerged as a novel class of RNAs with critical regulatory functions. However, a study on circRNAs during the floral transition of a crop plant is lacking. We investigated the expression and potential function of circRNAs in floral fate acquisition by soybean shoot apical meristem in response to short-day treatment.

**Results:**

Using deep sequencing and *in-silico* analysis, we denoted 384 circRNAs, with 129 exhibiting short-day treatment-specific expression patterns. We also identified 38 circRNAs with predicted binding sites for miRNAs that could affect the expression of diverse downstream genes through the circRNA-miRNA-mRNA network. Notably, four different circRNAs with potential binding sites for an important microRNA module regulating developmental phase transition in plants, miR156 and miR172, were identified. We also identified circRNAs arising from hormonal signaling pathway genes, especially abscisic acid, and auxin, suggesting an intricate network leading to floral transition.

**Conclusions:**

This study highlights the gene regulatory complexity during the vegetative to reproductive transition and paves the way to unlock floral transition in a crop plant.

**Supplementary Information:**

The online version contains supplementary material available at 10.1186/s12870-023-04319-3.

## Background

Soybean is an economically important crop as it is the primary oilseed source and high-quality plant protein for the human diet and livestock feed. Global soybean production has increased about 13-fold from 1961 to 2017, mainly due to the increased cultivation of arable land [1]. While the natural resources are limited, the demand for soybean and soybean products is rising. Thus, there is an urgent need to use modern crop breeding techniques to develop high-yielding soybean cultivars. Floral transition, or the switch from vegetative to reproductive growth, is a critical developmental stage in a plant life cycle, which ensures reproductive success and seed yield [2]. Further, soybean with paleopolyploid genome arising from two whole-genome duplication events contains multiple copies of flowering genes. Understanding the molecular mechanisms is the first essential step for developing high‐yielding crops and ensuring food security for the increasing world population, especially in changing climate.

The floral transition occurs in the shoot apical meristem (SAM), a multicellular dome-shaped structure located at the shoot apex. It contains a small population of undifferentiated and pluripotent stem cells. Endogenous signals and environmental factors regulate SAM growth and differentiation into organs such as stems, leaves, and flowers [3]. Photoperiod or day length is a critical environmental factor in regulating SAM development by extending the vegetative growth or inducing floral transition [4, 5]. In Arabidopsis, a facultative long-day plant, environmental cues (temperature and photoperiod) integrate endogenous signals (phytohormones and plant age) to regulate floral pathway integrator genes such as FLOWERING LOCUS T (FT) and SUPPRESSOR OF OVEREXPRESSION OF CONSTANS 1 (SOC1). Regulation of these integrators then activates floral meristem identity genes such as APETALA1 (AP1), FRUITFULL (FUL) and CAULIFLOWER (CAL) which leads to SAM floral transition and flowering [6]. Photoperiod is also a critical factor that determines soybean flowering and seed yield. Soybean cultivars are classified into maturity groups based on the photoperiod requirement. Soybean is a typical short-day plant, i.e., long-day photoperiod extends the duration of vegetative growth, and short days promote floral transition. The transition of soybean SAM from vegetative to reproductive is visible after six short days of treatment [7]. At the molecular level, short-day treated soybean SAM displayed significant changes in gene expression profile, mostly in genes related to several members of the MADS-box transcription factor family and genes associated with hormones such as auxin, abscisic acid, and jasmonic acid [8, 9]. Studies also reported the expression and potential function of non-coding RNAs such as microRNAs (miRNAs) and long non-coding RNAs (lncRNAs) in crop flowering [10] and in soybean SAM [11, 12]. For example, the expression of 277 lncRNAs associated with floral transition observed in short-day treated soybean SAM [12] suggests the role of regulatory RNAs in flowering. However, a study on circular RNAs is lacking.

CircRNAs are a distinct class of regulatory molecules, adding another layer of gene expression complexity in many eukaryotes [13]. CircRNAs are a form of non-canonical RNA molecules that splicing machinery generates from precursor mRNA using a special kind of splicing termed back-splicing. Through back-splicing, a downstream 3′ splice site joins an upstream 5′ splice site to form a covalently closed circle RNA with no 5′ or 3′ polarities [14]. Although most circRNAs have relatively low expression compared with their linear counterparts, they can play important roles in multiple biological processes in a tissue- or developmental stage-specific manner [15–17]. Whilst the role of circRNAs is becoming increasingly apparent in human development, and diseases [18], very little is known about their role in plants. Recent studies in plants suggested that circRNAs are closely associated with growth, development and response to external stimuli [19, 20].

To advance our knowledge of circRNAs during floral transition in soybean, we treated soybean plants with short-day photoperiod (8 h light/16 h dark) to induce the floral transition. We used micro-dissected SAM for RNase-R enriched RNA sequencing libraries. Using four different bioinformatics prediction tools, we identified 384 circRNAs expressed in SAM at four-time points of short-day treatment. Further, the back-splicing junction of 26 selected circRNAs was experimentally validated. Differential gene expression analysis and functional enrichment annotation highlighted the function of circRNAs during the floral transition, especially circRNA production from genes related to hormonal signalling pathways, abscisic acid, and auxin. Further, miRNA–circRNA interactions highlighted the potential role of circRNAs as competing-endogenous RNAs (ceRNAs) in post-transcriptional gene regulation.

## Results

### Identification and characterization of 384 circRNAs in soybean SAM

**Fig. 1:**
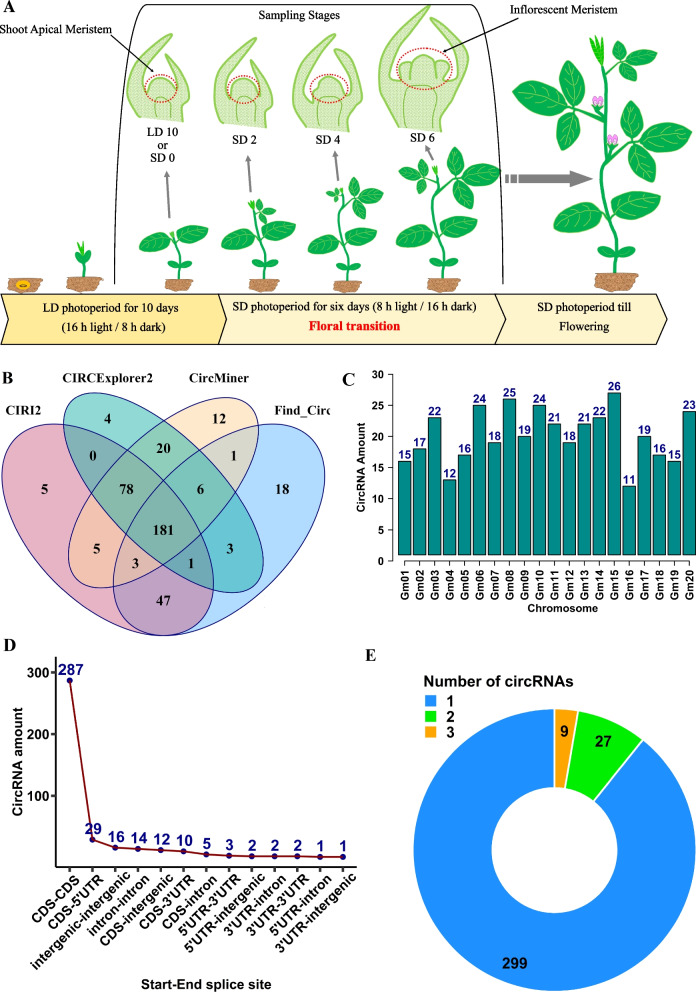
Schematic representation of sampling during soybean floral transition and features of identified circRNAs in soybean shoot apical meristem. **A** Plants were grown for 10 days under long-day (LD) photoperiod (16 h light/8 h dark), and then transferred to short days (SD) (8 h light/16 h dark). In soybean, flowering occurs when plants expose to short day photoperiod, but floral transition generally induced within the first six days of short-day treatment. Red doted circles illustrate shoot apical meristem that were dissected from plants (SD 0, SD 2, SD 4, and SD 6). Stages of growth based on nomenclature in (Fehr & Caviness, 1977): VE: emergence, VC: unrolled unifoliolate leaves, V1: first trifoliolate, V2: second trifoliolate, and R1: beginning flowering. **B** Venn diagram of unique and common circRNAs predicted by four detection tools. Of 384 identified circRNAs, 181 were common among identification tools. **C** Distribution of circRNAs on chromosomes. CircRNAs are distributed unevenly on different chromosomes. Chromosome 16 generated the most, and chromosome 11 generated the least amount of circRNAs. **D** The number of circRNAs generated from each genomic region. The vast majority of circRNAs overlapped entirely or partially with genic regions. **E** The parent genes of circRNAs produced different numbers of circular transcript isoforms. Of 335 circular producing genes, 299 generated only one circRNA isoform

To investigate the potential roles of circRNAs during floral fate acquisition by soybean SAM, circRNA-enriched RNA sequencing libraries (RNase R treated libraries for removing linear RNAs) were constructed for four short-day (SD) conditions: SD 0, SD 2, SD 4, and SD 6 with three biological replicates (Fig. 1A). RNA sequencing experiment produced over 1.4 billion clean reads with Q20 of about 98% for all 12 biological samples (Additional file 1: Table S1). Analysis of the RNA sequencing data with four circRNA detection tools identified a total of 2580 circRNAs in all treatments with the criteria that a circRNA must be present in three biological replicates and supported by at least two back-spliced reads (Additional file 1: Table S2). A total of 384 unique circRNAs were obtained by combining common circRNAs identified in different treatments and their biological replicates (Additional file 1: Table S3). To pinpoint those specifically expressed in SAM tissue, we compared the circRNAs identified in this study with published soybean circRNAs from root, stem, and leaf tissues [21]. Out of the 384 circRNAs analysed, 259 were found to be exclusively expressed in SAM tissue, while the remaining 125 were expressed in other tissues (Additional file 1: Table S3). When comparing the number of circRNAs found by different detection tools, 320, 306, 293, and 260 circRNAs were found by CIRI2, CircMiner, CIRCExplorer2, and find_circ software respectively, and 181 circRNAs were common between four identification tools (Fig. 1B).

The distribution of circRNAs among chromosomes showed uneven transcription of circRNAs from different chromosomes; chromosome 15, with 26 circRNAs, generated the most, and chromosome 16, with 11 circRNAs, generated the least amount of circRNAs (Fig. 1C). Moreover, the annotation analysis revealed that circRNAs could arise from diverse genomic regions, including Coding DNA Sequences (CDS), 5´ and 3´ Untranslated Regions (UTR), as well as intronic and intergenic regions (Fig. 1D, Additional file 1: Table S3). Among 384 identified circRNAs, 92.19% (354) of circRNAs contain at least one exon from coding sequences, 4.17% were intergenic, and 3.64% were from intronic regions (Fig. 1D). While most of the circRNA-producing genes could generate only one isoform of circRNAs, 36 genes were able to produce two or three isoforms of circRNAs through alternative back-splicing (Fig. 1E). We also noted ten circRNAs generated from two or three adjacent genes (Additional file 1: Table S3).

### Experimental validation of circRNAs using divergent primers and Sanger sequencing

**Fig. 2:**
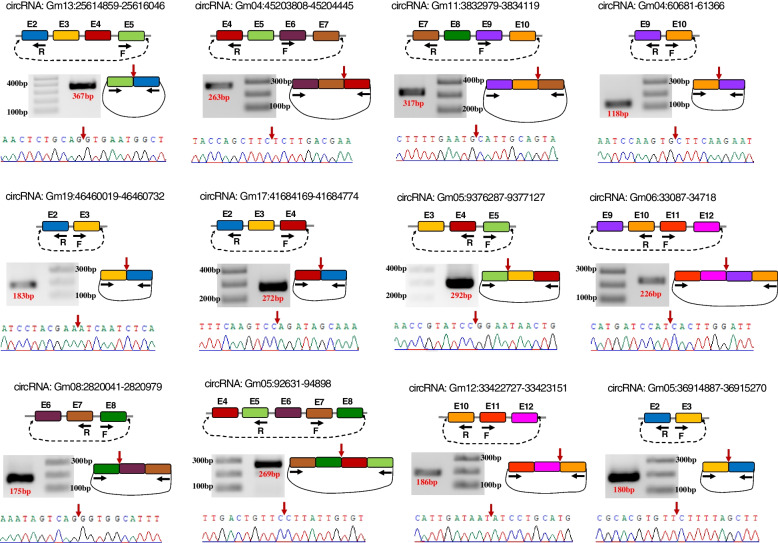
Validated circRNAs in soybean shoot apical meristem using divergent primers and Sanger sequencing. In each section, from top: circRNA ID; the exons (E) involved in circRNA; black arrows representing the position of forward (F) and reverse (R) primers; the amplified circRNA junction and its size on agarose gel (1%, TAE buffer); red arrow representing the position of circRNA junction; confirmed circRNA junction sequence by Sanger sequencing. Full-length gel images are presented in Additional file 5: Note S1

To confirm the authenticity of the identified circRNAs, we selected 30 circRNAs whose parent genes or their miRNA targets were predicted to be involved in floral transition in soybean SAM. A set of divergent primers listed in Additional file 1: Table S4 were designed for amplifying the back-splicing junction of each selected circRNA. Twenty-six circRNAs were successfully validated using PCR with divergent primers. We further checked the accuracy of the back-splicing sites by using 50% of amplified circRNA [13] to Sanger sequencing. The results verified the junction sequence of all the samples (Fig. 2; Additional file 5: Note S1). It is worth noting that all the validated circRNAs were made of only exons with introns spliced out.

### Exonic circRNAs contain multiple exons, and their overall length is under 1000 nucleotides

**Fig. 3:**
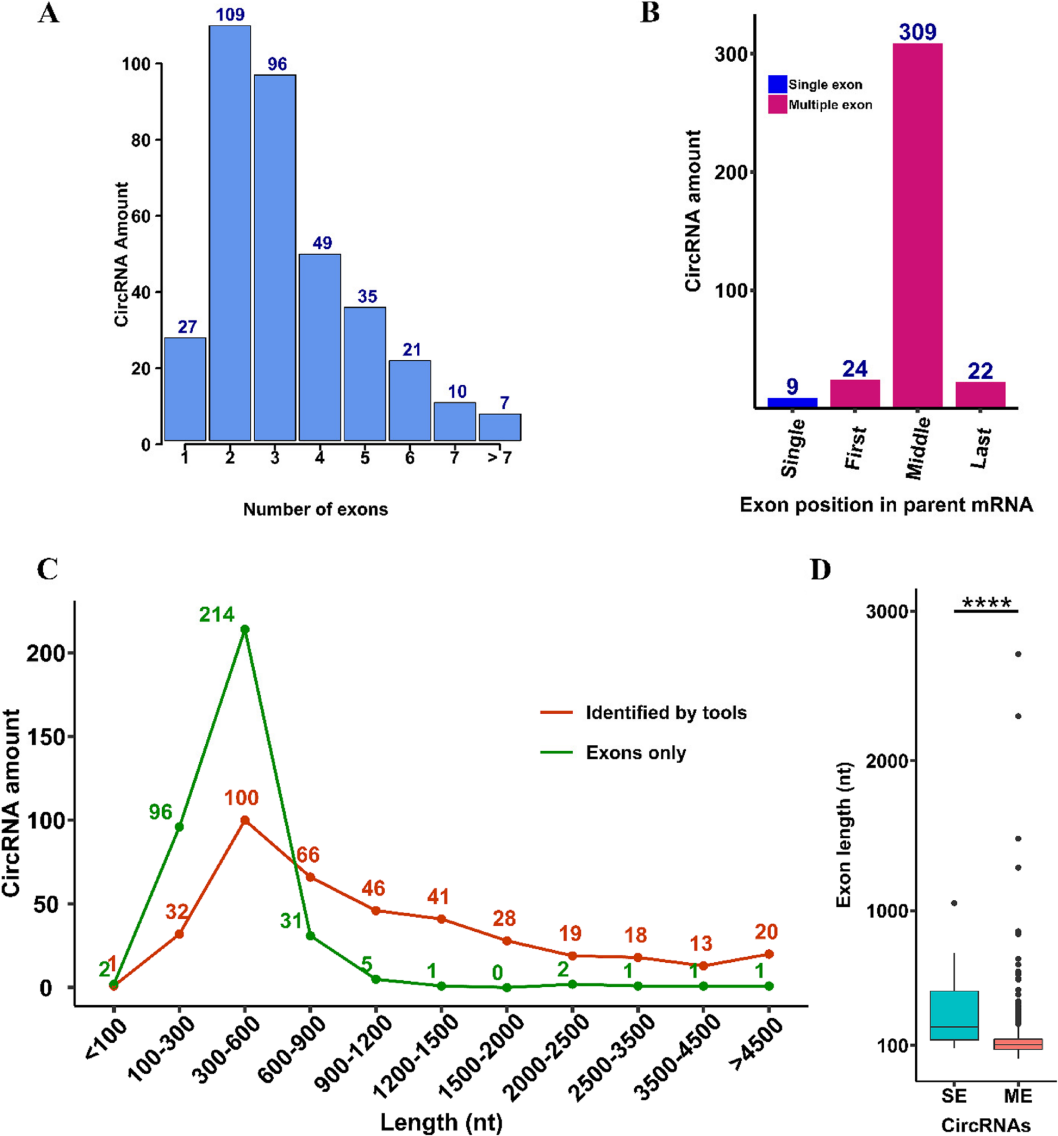
Features of exonic circRNAs expressed in soybean shoot apical meristem. **A** The number of exons in each circRNA. CircRNAs were mainly composed of two to four exons. **B** The position of exons in the parent gene of circRNAs. A large number of circRNAs originated from the middle exons of their parent genes. **C** Length distribution of circRNAs. Red colour: the length of circRNAs originally identified by detection tools. Green colour: the length of exonic circRNAs after removing introns. The peak length was between 300 to 600 nucleotides (nt). **D** Comparison of exon length between single-exon (SE) and multiple-exon (ME) circRNAs. SE circRNAs contain significantly longer exons compared with ME circRNAs (****p value = 2.1e^−9^, Wilcoxon rank-sum test)

Further analysis of our identified circRNAs revealed that exonic circRNAs were composed of one to 14 exons, but most (66.15%) contained two to four exons (Fig. 3A). The exons in multiple-exon circRNAs mainly originated from the middle exons (84.89%) of their annotated genes rather than the first or last exons; nine circRNAs were also found to be originated from the single-exon genes (Fig. 3B). The length distribution of the identified circRNAs was between 100 to 1500 nucleotides (nt). When considering the length of exons in exonic circRNAs, the length distribution was mainly between 100 to 900 nt, with only a few circRNAs longer than this range (Fig. 3C). We also compared the length of exons between single-exon circRNAs and multiple-exon circRNAs. The results showed that single-exon circRNAs contained exons that were significantly longer than exons in multiple-exon circRNAs (Fig. 3D).

### Reverse complementary sequences in the flanking introns could promote circRNA biogenesis in soybean SAM

**Fig. 4:**
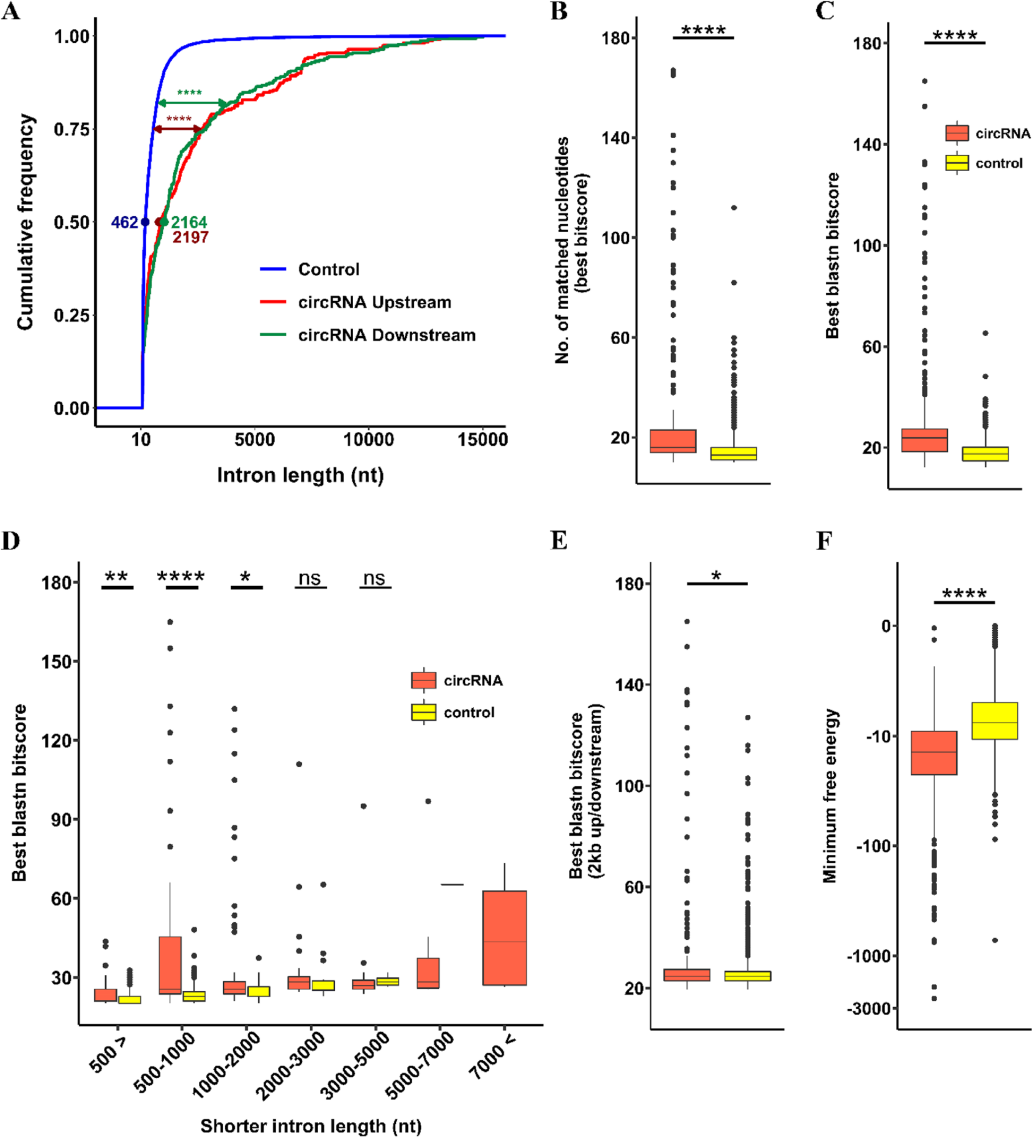
Features of introns flanking circRNAs. Wilcoxon rank-sum test was used as a statistical test in all sections. **A** Length distribution of upstream (red) and downstream (green) flanking intron of circRNAs compared with flanking intron of control exons (blue). Flanking introns of circRNAs were significantly longer than controls (****p value < 2e^−16^). **B** The number of complementary nucleotides found between upstream and downstream introns of circRNAs and controls. Flanking introns of circRNAs contained much more complementary nucleotides than controls (****p value < 2e-16). **C** Comparison of best BLAST scores between flanking introns of circRNAs and controls. Flanking introns of circRNAs had significantly higher scores which mean stronger alignment between identified complementary sequences (****p value < 2e-16). **D** Length distribution of flanking introns and their BLAST score. Flanking introns of circRNAs had higher BLAST scores compared with controls, especially in the ranges below 500 up to 1000 nucleotides (**p-value 0.00613, ****p value 1.6e^−8^, *p-value 0.01314). **E** Comparison of best BLAST scores between flanking sequences of circRNAs and controls. Here the flanking sequences were selected from the immediate 2000 nucleotides upstream and downstream of circRNAs and controls. Flanking sequences of circRNAs had slightly higher scores than controls (*p-value 0.0048). **F** Minimum free energy of identified complementary sequences in the flanking intron of circRNAs and controls. Complementary sequences in the flanking intron of circRNAs had significantly lower minimum free energy, which means stronger base paring (****p value < 2e-16). In sections **B**, **C**, **D**, and **E**, numbers above 180 on the vertical axis were used in the statistical test but not shown in the plots for graphical reasons

Next, we compared the length of introns flanking circRNAs with the length of introns flanking a list of simulated lists of control exons prepared as detailed under the materials and methods section. The results showed that circRNAs were flanked by significantly longer introns in their upstream and downstream regions (Fig. 4A). The BLAST command line was used to scan upstream and downstream introns of circRNAs and control exons to check the presence of reverse complementary sequences (Additional file 2: Table S5). The number of complementary bases and the best BLAST score (bitscore) were significantly higher in flanking introns of circRNAs compared with the control list (Fig. 4B and C). As this higher statistical significance could be just the result of longer introns that flank circRNAs, statistical analysis was used to test the BLAST score against the length of flanking introns. The results revealed that in some ranges of intron length, especially from below 500 nt up to about 1000 nt, flanking introns of circRNAs contain a significantly higher amount of reverse complementary sequences compared with controls (Fig. 4D). The comparison at longer intron size (above 5000 nt) was not possible as introns flanking controls were not in that size range. We also explored the presence of reverse complementary elements in the immediate 2 kb of the flanking sequences (not necessarily introns) of circRNAs and controls (Additional file 3: Table S6). The result still displayed a higher BLAST score in flanking sequence of circRNAs, but with less degree than the flanking intron results (Fig. 4E). To further investigate the flanking introns of circRNAs, the minimum free energy (MFE) of the identified reverse complementary sequences in the flanking introns of circRNAs and controls were compared. The results revealed significantly lower MFE in flanking introns of circRNAs which means stronger base-pairing between upstream and downstream reverse complementary elements (Fig. 4F). All these results suggest that reverse complementary sequences in the flanking introns of circRNAs might play a role in circRNA biogenesis in soybean SAM.

### CircRNAs have distinct expression patterns in soybean SAM in response to short-day treatment

**Fig. 5:**
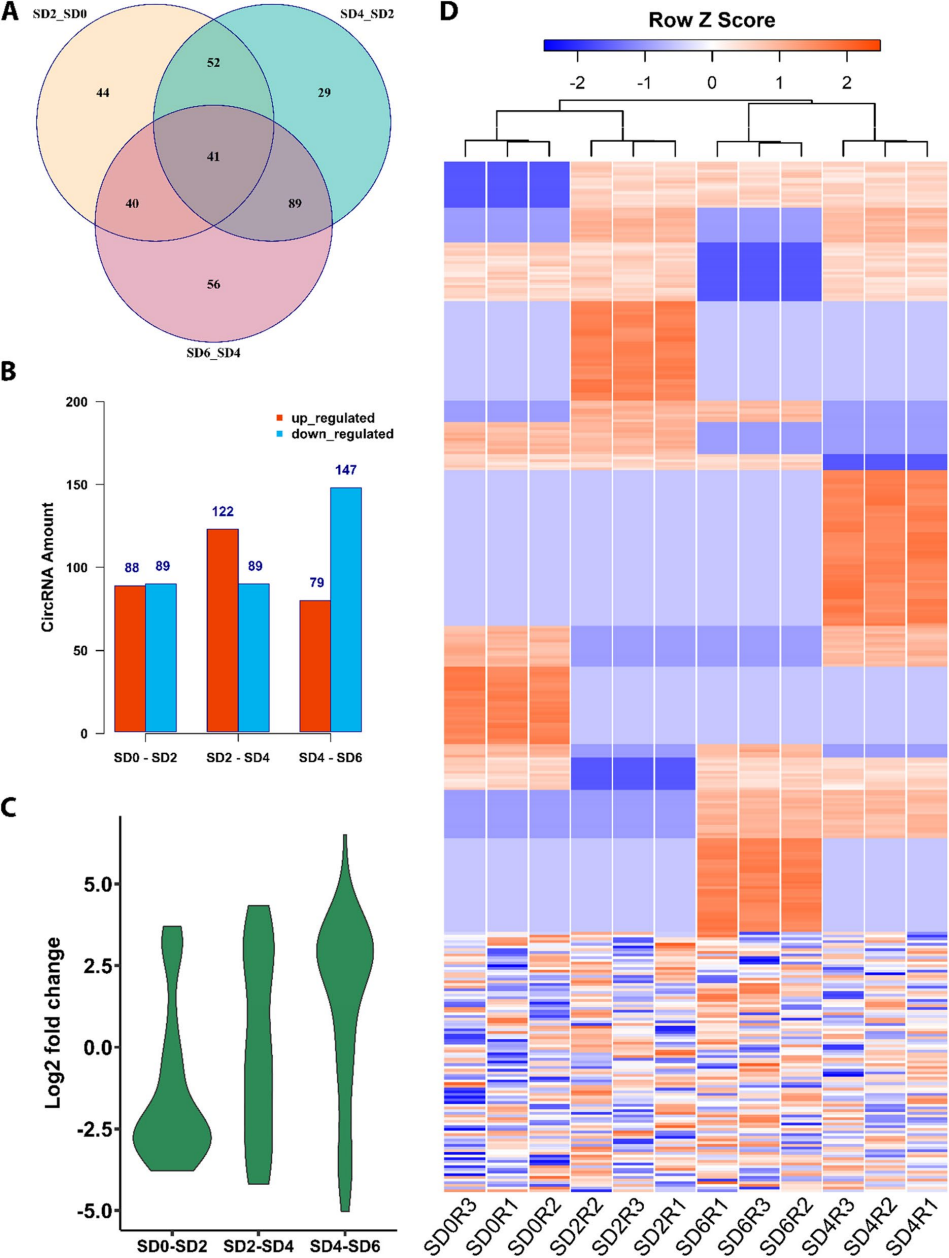
Expression pattern of circRNAs in soybean shoot apical meristem. **A** Venn diagram illustrates the number circRNAs with differential expression in response to short-day photoperiod treatment. Of 351 differentially expressed circRNAs, 129 showed treatment-specific expression patterns, while 41 were expressed commonly at all time-point of photoperiod treatment. **B** Histograms represent the number of up- or down-regulated circRNAs responding to photoperiod treatment. **C** The Violin plot illustrates the expression pattern of 129 short-day treatment specific circRNAs. The expression pattern of circRNAs changed when the plants were exposed to different time-point of photoperiod treatment. While the expression of treatment-specific circRNAs down-regulated when plants transferred to short-day photoperiod, the expression of circRNAs up-regulated after six short-day of photoperiod. **D** The heatmap shows the expression profile of 384 identified circRNAs in all samples with their biological replicates

To explore the expression pattern of circRNAs during floral transition in soybean SAM, we compared the expression profile of circRNAs in each photoperiod time-point with its previous time-point (Additional file 4: Table S7). The results revealed differential expression of 351 circRNAs, of which 129 were expressed explicitly in response to different photoperiod treatments (Fig. 5A). The overall number of differentially expressed circRNAs tended to up-regulate following four short days and down-regulate after six short days (Fig. 5B). However, when we visualized the expression pattern of 129 circRNAs specifically expressed in response to photoperiod treatment, we observed that in the early short-day treatment, more circRNAs showed down-regulated expression, and in short-day six, upregulation was more prevalent (Fig. 5C). We also visualized the expression profile of circRNAs vs. different photoperiod treatments and their biological replicates using a clustering heatmap (Fig. 5D). The expression analysis results indicated that circRNAs have a distinctive expression pattern in response to photoperiod treatment, suggesting their potential roles in floral transition of soybean SAM.

## Differentially expressed circRNAs contain one to four miRNA binding sites

**Fig. 6:**
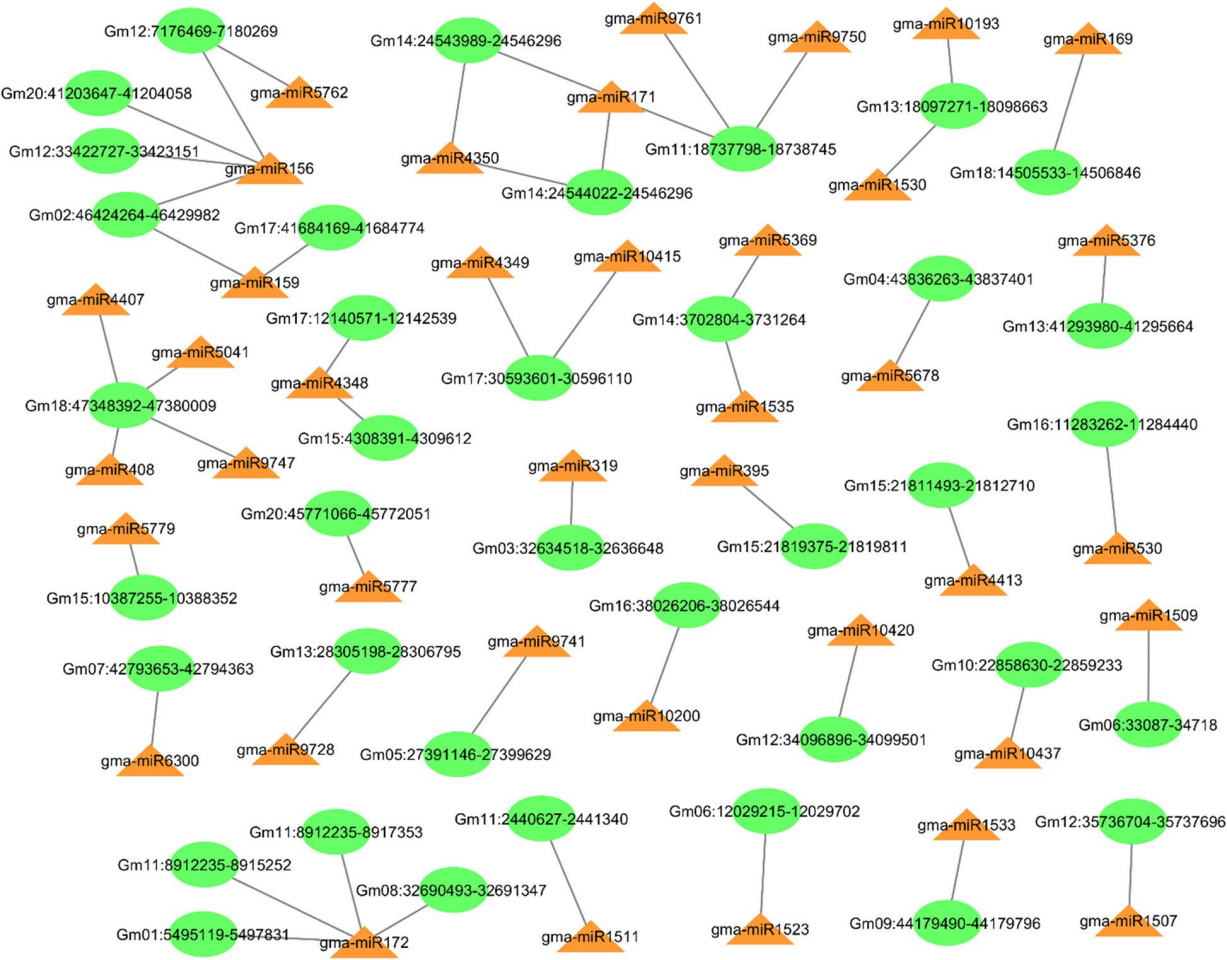
Predicted circRNA-miRNA interactions for differentially expressed circRNAs in soybean shoot apical meristem. CircRNAs could potentially contain one to four miRNA binding sites

To further evaluate the function of circRNAs in post-transcriptional gene regulation during floral transition in soybean SAM, we predicted the miRNA binding sites on differential expressed circRNAs (Additional file 4: Table S8). The results revealed 38 circRNAs with putative binding sites for 39 miRNAs. Most circRNAs (29 out of 38) could target only one miRNA, and we found seven circRNAs that contained two potential miRNA binding sites. We also noted that circRNA Gm11:18737798-18738745 and Gm18:47348392-47380009 could target three and four miRNAs, respectively (Fig. 6). Similarly, miRNAs could be targeted by a different number of circRNAs. Of 39 identified miRNAs, 33 had only one binding site on circRNAs, and six were targeted by two to four circRNAs (Fig. 6). For example, Gma-miR156 and Gma-miR172 were the miRNAs that four circRNAs could target. These results suggest that circRNAs might be involved in gene regulation at the post-transcriptional level in soybean SAM.

### CircRNAs function during floral transition through miRNA sponging

**Fig. 7:**
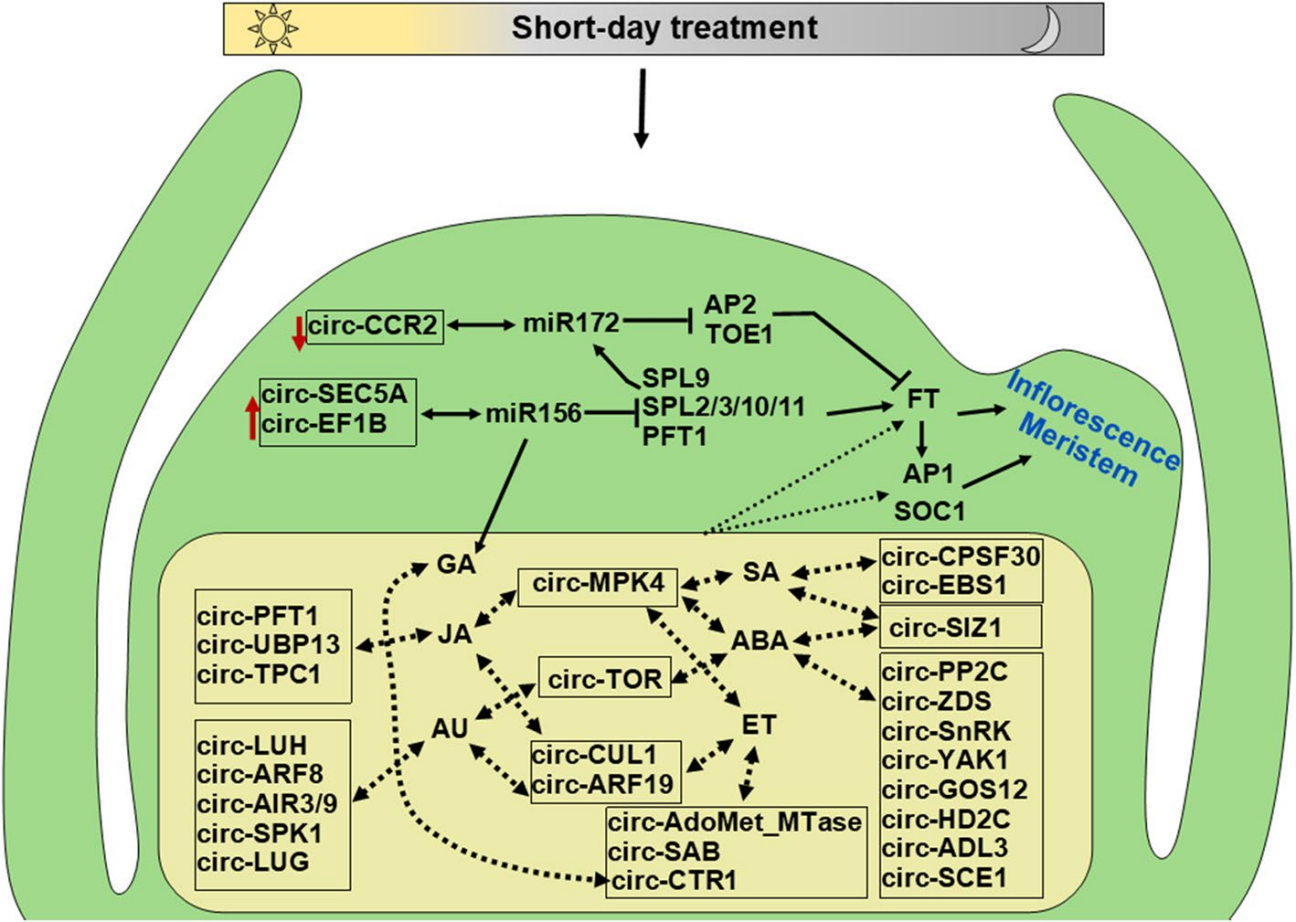
Proposed interaction of circRNAs with miR172 and miR156, and hormonal signaling pathway in shoot apical meristem of soybean. During the floral transition, the downregulation of circ-CCR2 increases the availability of miR172, leading to the downregulation of AP2 and TOE1 and the upregulation of FT. On the other hand, upregulation of circRNAs that sponge miR156, such as circ-SEC5A and circ-EF1B, decreases the availability of this miRNA, resulting in increased expression of SPLs and PFT1, which promotes expression of FT. Genes related to the hormonal signaling network showed circRNA production activity. These circRNAs might be involved regulating flowering genes and floral transition via interacting with hormonal signaling pathways through unknown mechanisms. Red arrows represent the upregulation or downregulation of specified circRNAs. Dashed arrows represent the possible interaction of circRNAs with hormonal signaling and flowering genes. Genomic position and properties of circRNAs are in Additional file 1: Table S3

In our experiments, we further explored the target genes of well-known miRNAs, miR172 and miR156. MiR172 could target 164, and miR156 could target 199 downstream genes, mainly related to meristem development and flowering. For example, soybean genes Glyma.11G117300 and Glyma.11G117400, which are involved in the vegetative to the reproductive phase transition of the meristem, were able to produce an exonic circRNA from the genomic region Gm11:8912235-8915252 (circ-GmCCR2). Circ-GmCCR2 had downregulated expression in short-day treated plants and could potentially target miR172. Downregulation of circ-GmCCR2 can lead to upregulation of FLOWERING LOCUS T (FT) and initiation of floral transition as miR172 target genes such as Gm-AP2 and Gm-TOE1, which negatively regulate the expression of GmFT. We also noted two circRNAs with upregulated expression trends in short-day treated samples, which could further promote floral transition through sponging miRNA 156 (Fig. 7).

### CircRNAs modulating hormonal pathways and transcriptional regulation during the floral transition

Since plant hormones regulate floral transition and flowering [22], here we also investigated the expression of circRNAs from genes related to different hormonal pathways. The result revealed 40 genes with circRNA production activity with various hormonal pathways, including abscisic acid, auxin, jasmonic acid, salicylic acid, and ethylene. CircRNAs generated from these genes could potentially regulate biosynthesis or the signaling pathway of one or multiple hormones through a potentially complex interaction network leading to floral transition (Fig. 7). In addition, we conducted a more detailed exploration of the potential role of circRNAs in gene regulation, specifically through their involvement in transcription. Interestingly, we found that 23 of the identified circRNAs are derived from genes that function as transcription factors, including Phytochrome and Flowering Time 1, Auxin Response Factor 8, Embryonic Flower 2, Flowering BHLH 3, Reduced Vernalization Response 2, and Basic Region/Leucine Zipper Transcription Factor 16 (Fig. 7, Additional file 1: Table S3). This suggests that circRNAs may play a significant role in regulating gene expression at the transcriptional level.

### Functional annotation analysis shows circRNAs can regulate meristem development and floral transition in short-day treated soybean SAM

**Fig. 8:**
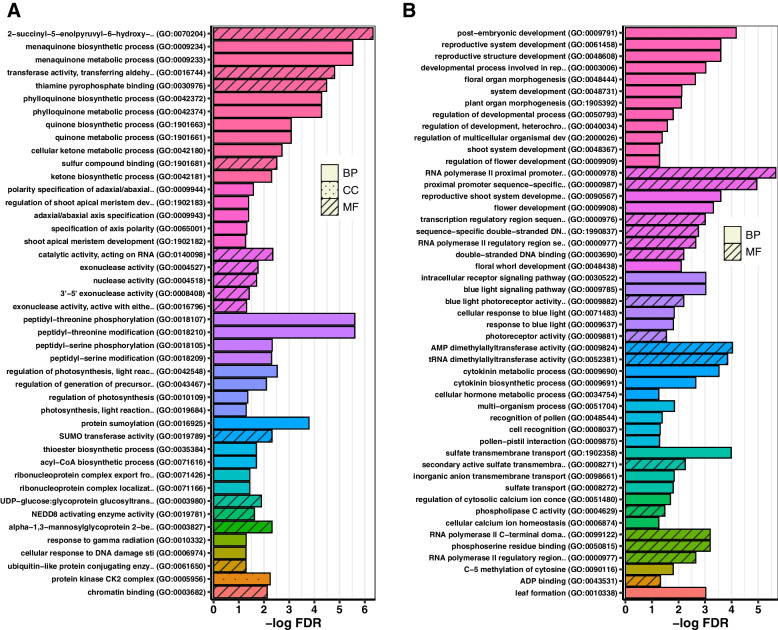
Functional enrichment analysis of circRNA parent genes (**A**) and their miRNA target (**B**). The enriched Gene Ontology (GO) terms for parent genes of circRNAs were mainly related to protein modification, photosynthesis, and meristem development, while for miRNA targets, GO terms were mainly related to flowering and reproduction. Bars with the same colour represent GO groups, as summarized by ClueGO

To investigate the potential functions of circRNAs during floral transition in soybean SAM, we performed GO enrichment analysis on the parent genes of differential expressed circRNAs and their miRNA target genes. The GO terms belonged to biological process, molecular function, and cellular component categories (Additional file 4: Table S9). For the parent genes of circRNAs, enriched GO terms were mainly related to protein modification and meristem development. For example, adaxial/abaxial axis specification, shoot apical meristem development, and specification of axis polarity were enriched GO terms related to meristem development (Fig. 8A). To investigate the potential function of miRNA targets, we first predicted the target genes of 39 miRNAs that had binding sites on circRNAs, and the results showed that miRNAs could target more than 2300 unique genes (Additional file 4: Table S10). The enriched GO terms of miRNA target genes belonged to various processes but were mainly related to reproduction and flowering. For instance, reproductive shoot system development, the developmental process involved in reproduction, and regulation of flower development were the GO terms that enriched the biological process category (Fig. 8B; Additional file 4: Table S11). We observed that, among the 129 circRNAs that were specifically expressed during the short-day treatment (Fig. 5C), the parent genes of downregulated circRNAs in the early stage of short-day treatment were mostly enriched in various metabolic processes, such as primary metabolic process, organic substance metabolic process, cellular macromolecule metabolic process, and nitrogen compound metabolic process. In contrast, during the later stages of the short-day treatment, the upregulated parent genes of circRNAs belonged to GO terms involved in the regulation of cell morphogenesis, cell shape, organelle localization, and regulation of anatomical structure morphogenesis. The functional annotation results suggest that circRNAs may play a role in regulating these processes during floral transition. These results suggest that circRNAs could be involved in diverse biological and molecular processes during floral transition in soybean SAM via direct regulatory functions or by targeting miRNAs in post-transcriptional regulation.

## Discussion

CircRNAs are a distinct class of regulatory molecules that continuing scientific research, especially over the past decade, categorized them as another layer of gene expression complexity in many eukaryotes including several plant species. Although most circRNAs have relatively low expression compared with their linear counterparts, they can play important roles in multiple biological processes in a tissue- or developmental stage-specific manner [15, 16, 23]. Whilst the role of circRNAs is becoming increasingly apparent in human development and diseases [18], very little is known about their role in plants. Recent studies in plants suggested that circRNAs are closely associated with growth and development as well as response to external stimuli [19, 20]. In the present study, we explored the potential role of circRNAs in floral fate acquisition by soybean shoot apical meristems (SAM) under short-day photoperiod conditions. In total, we identified the expression of 384 circRNAs in soybean SAM in all short-day photoperiod treatments. We observed that the back-splicing sites in more than 74% of circRNAs were located within the CDS at both ends. Of 335 circRNA producing genes, more than 89% generated only one isoform of circRNAs, and the remaining were able to produce two or three isoforms. We also found 10 circRNAs that were generated from two or three different genes. Most of our identified circRNAs in soybean SAM were constituted of one to four exons, and the vast majority of them were originated from the middle exons of their parent genes. Our study identified 351 circRNAs which showed differential expression patterns in soybean SAM at different time-points of short-day photoperiod treatments. We found 129 circRNAs that had specific expressions in response to photoperiod with high up-regulation expression after six days of short-day treatment. These results suggest that circRNAs might be involved in the regulation of growth and development processes in soybean SAM in response to photoperiod.

In plants, circRNAs could be involved in developmental processes such as floral transition and flowering. For instance, a naturally occurring circRNA derived from exon 6 of SEPALLATA3 (SEP3; a gene known to be involved in flower development) was required for normal floral organ development in Arabidopsis. Overexpression of SEP3 circRNA in Arabidopsis resulted in changes in flower phenotypic features such as fewer stamen and additional petals in transgenic lines compared with wild type [24]. During fertility transition and pollen development, circRNAs were also found to be involved in the regulation of pollen-related developmental processes such as cell cycle and cell division, cell differentiation, and floral organ development [17]. Flowering in soybean is a complex process as each flowering gene found in Arabidopsis could have multiple orthologous copies in soybean [25–27]. Previous studies have shown that the gene expression in soybean SAM changes significantly during the floral transition with the potential function in several processes such as biosynthetic and metabolic process, regulation of cell size, and flower development [28]. Histone modifiers and RNAi-associated genes have also been reported to have high expression in soybean SAM during the floral transition [29]. As circRNAs may function in processes related to the function of their parent genes, we performed GO enrichment analysis on parent genes of differential expressed circRNAs to better understand the potential function of circRNAs during floral transition in soybean SAM. Similar to the previous studies, we noted that the parent genes of circRNAs might play roles in diverse biological processes but mainly in processes related to photosynthesis, protein modification, and meristem development. For example, phylloquinone (vitamin K1) is required for photosynthetic electron transport in the photosystem I redox chain [30]. Vitamin K is also an essential co-factor for enzymes that modify proteins in post-translational events [31]. Polarity specification of adaxial/abaxial axis and regulation of shoot apical meristem development were examples of enriched GO terms related to meristem development in our analysis. Based on our observation, we speculated that circRNAs might be involved in processes related to protein modification that could lead to the regulation of SAM growth and its floral transition in soybean.

Previous studies in animals and humans have revealed that circRNAs can act as miRNA sponges suggesting their potential function as post-transcriptional gene expression regulators. Typical examples of such circRNAs are circSry, with 16 binding sites for miR-138 in mice testis and CDR1as has 70 binding sites for miR-7 in the mammalian brain [32, 33]. Studies have shown that when the expression of CDR1as is reduced in human cells, the level of mRNAs with binding sites for miR-7 decreases indicating that the CDR1as can play its role in sponging miR-7 [32, 33]. Plant circRNAs could target miRNAs to regulate gene expression at the post-transcriptional level [34–37]. For example, in Arabidopsis, a circRNA named circ_At3g13990 has the potential to form a miR4239-5p- argonaut-circRNA ternary complex to downregulate the expression of target genes that have high expression in carpels and flowers [38]. In *Brassica campestris*, it has been suggested that upregulation of a circRNA derived from the A02:23507399|23531438 locus could suppress a few miRNAs that target Bra002750 gene (involved in the regulation of several pollen development-related processes), resulting in up-regulation of this gene and production of abortive pollen [39]. Also, a recent study using the CRISPR-Cas9 system in rice showed that a circRNA named Os06circ02797 could sponge and sequester Os-miR408 yielding faster-growing mutant seedlings with higher chlorophyll content compared with wild-type plants [40].

Herein, we found that 38 differential-expressed circRNAs could potentially contain one to four putative binding sites for 39 miRNAs. Among the predicted miRNAs, we noted some important and well-studied miRNAs, such as miR156, miR159, miR169, miR171, miR172, miR319, and miR395 which all are involved in biological processes regulation during growth and development in plants including flowering [41–43]. For example, the expression level of miR156 and miR172 regulates the developmental phase transition in plants [44, 45]. At the early stages of plant growth, miR156 expresses at the highest level while miR172 has a low expression; as plants age, the expression level of miR156 decreases and the level of miR172 increases, resulting in the transition from vegetative to reproductive growth [46]. MiR156 targets and restricts genes related to stage transition and flowering time, such as SQUAMOSA PROMOTER BINDING PROTEIN-LIKE (SPL) genes, but miR172 restricts the expression level of APETALA 2 (AP2) genes which regulate floral transition, and flower meristem identity [45, 47]. Our analysis found four circRNAs that could target miR172 and four circRNAs with potential binding sites for miR156. One example in our analysis is circRNA Gm20:41203647-41204058 (circ-SEC5A), which is upregulated following the short-day photoperiod treatment. The up-regulation of this circRNA can potentially attenuate miR156 levels leading to up-regulation of the downstream flowering-related genes such as SPL9 to promote the floral transition in soybean SAM. SPL9 can activate the expression of miR172 and further promote flowering [47, 48]. Based on our results and earlier findings, we suggest that circRNAs have the potential to play vital functions through the ceRNAs network during SAM development and floral transition in soybean.

It is known that hormonal signalling and interaction between different hormones regulate the flowering time and floral transition in plants [22, 49]. Plant hormones can constitute a complex signalling pathway in response to endogenous and exogenous factors such as plant age, and photoperiod to modulate various developmental stages including flowering [50]. While gibberellin is one of the well-studied phytohormones in relation to flowering, the contribution and role of other hormones, such as abscisic acid, jasmonic acid, salicylic acid, and ethylene, have also been reported [50]. For example, ABA is generally known as the drought-stress-induced hormone. Still, it’s positive regulatory effect on floral transition has been revealed, particularly via the activation of flowering genes such as CONSTANS (CO) and FT [51, 52]. Salicylic acid has also been reported to be involved in floral transition by regulating flowering genes such as FT, SOC1, and CO [53]. The transcription and regulation of multiple hormonal genes such as gibberellin, abscisic acid, jasmonic acid, and auxin have also been reported previously in soybean during floral transition in response to short-day treatment [9, 28]. Here in this study, we observed that genes related to hormonal pathways actively produce circRNAs in soybean SAM during the floral transition, especially genes related to abscisic acid and auxin. For instance, PROTEIN PHOSPHATASE TYPE 2C (PP2C) and SUCROSE-NON-FERMENTING-1-RELATED PROTEIN KINASE-2S (SnRK2) are the core components of ABA signalling in plants. We found that GmPP2C (Glyma.02g162300) and GmSnRK3.23 (Glyma.15g197300) produced circRNAs that might regulate the expression of ABA signalling components. We also found some circRNAs that could function in the biosynthesis processes of hormones. For example, ZETA-CAROTENE DESATURASE (ZDS) is an enzyme that works in the carotenoid biosynthesis pathway, a substrate for abscisic acid production in plants. Although the functional mechanisms of circRNAs derived from hormonal signalling pathway genes need further research, based on models proposed in plants, circRNAs could function in the regulation of their parent gene transcription via interacting with RNA polymerase II, form R-loop and alter the splicing of their locus by promoting exon skipping, affect translation by outcompeting mRNAs for binding the same proteins, or even directly bind mRNAs and increase their stability and translation [13, 24].

## Conclusion

In conclusion, our findings highlighted the expression profile of circRNAs in soybean SAM in response to short-day photoperiod treatment. We report 384 circRNAs, and most had differential expression during floral transition. circRNA-miRNA network analysis suggested the potential function of circRNAs as gene regulatory elements at the post-transcriptional level. Functional enrichment analysis revealed the host gene of circRNAs, and miRNA targets are mainly related to protein modification and meristem development, especially flowering. Our study also highlights the role of circRNAs in modulating the expression of hormonal signalling genes in regulating floral transition. Our results pave the way for further research to unlock the complexity of gene regulation during floral transition.

## Materials and methods

### Plant materials, and sequencing

Soybean (*Glycine max*) plants, cultivar “Bragg” (non-transgenic), were grown under controlled conditions with a constant temperature of 25 °C, 400 μmol m^−2^ s^−1^ light intensity, and 70% humidity. The plants were initially grown for 10 days in the long-day (LD) photoperiod (16 h light/8 h dark), and then transferred to the short-day (SD) treatment (8 h light/16 h dark) for 2, 4, and 6 days (Fig. 1A) [54]. To collect samples, the shoot apical meristem (SAM) was micro-dissected from approximately 100 plants within a 2-h timeframe for each time-point: SD0 (LD10), SD2, SD4, and SD6, using a dissecting microscope at 40 × magnification, following the previously described method [55]. We excluded any leaf primordia to ensure that the collected tissue was enriched in meristem cells. The location of the collected SAM tissue is indicated in Fig. 1A. Three biological replicates for each time points were used. Dissected SAM samples were immediately frozen in liquid nitrogen and stored at −80 °C for later use.

Total RNA was isolated from SAM samples using the mirVana™ miRNA Isolation Kit (Thermo-Fisher; Part Numbers AM1560, AM1561, Carlsbad, CA, USA) according to the manufacturer’s instructions. TURBO™ DNase (Ambion, Carlsbad, CA, USA) was used to remove the remaining DNA from isolated RNA samples. The clean and high-quality RNA samples were used for circRNA sequencing (BGI Group, Hong Kong). The RNA-seq libraries were generated following RNase R treatment to remove linear RNAs. Paired-end read (100 bp) sequencing was performed using DNBseq Eukaryotic Transcriptome resequencing kit on DNBseq™ platform. The sequencing data were deposited to NCBI’s Sequence Read Archive (SRA) under accession number PRJNA893233.

### Identification, characterization, and differential expression of circular RNAs

To identify the expression of circRNAs in soybean SAM, we first mapped our RNA sequencing data against *Glycine max* reference genome (Wm82.a4.v1, Phytozome, https://phytozome-next.jgi.doe.gov/) [56, 57] using BWA (v0.7.17, mem-T 19) [58], Bowtie2 (v2.3.5.1) [59], STAR (v2.7.5a) [60], and CircMiner (v0.4.5) [61] with their default parameters. Mapping results were then analysed with four different circRNA detection tools: CIRI2 (v2.0.6) [62], find_circ (v1.2) [32], CIRCexplorer2 (v2.3.8) [63], and CircMiner (v0.4.5). After removing repeated circRNAs identified by two or more detection tools, a list of unique circRNA candidates with at least two supporting reads was generated for downstream analysis.

Using command lines from Bedtools (v2.30.0) [64], Bedops (v2.4.40) [65], and Genome-Tools (v1.6.2) [66], we extracted genomic features and properties of our identified circRNAs based on the corresponding genome annotation file obtained from Soybase (https://soybase.org/genomeannotation/) [67]. For comparison analysis, we used the aforementioned tools to create a list of probable linearly spliced exons as a control. The control list was created as follows: (i) a total of 10,000 genes (with the exons from only one mRNA isoform for each gene) were randomly selected from the soybean annotation file; all the circular producing genes identified in this study were removed from the selected genes; (ii) a diverse combination of exons from one exon up to six consecutive exons were extracted from the selected genes and saved as a list; (iii) similar to the portion of exon count in circRNAs found in this study (e.g., circRNAs that contain one exon, two exons, etc.), controls were randomly selected from the previous step list (Additional file 4: Table S12).

Basic Local Alignment Search Tool (BLAST, v. 2.9.0) [68] was used to find complementary elements in flanking introns of circRNAs and control exons, and RNAFold [69] was used to compute the minimum free energy of the identified complementary sequences.

To analyse the tissue specificity of circRNAs, we compared the sequences of the circRNAs identified in this study to those previously reported in soybean root, stem, and leaf tissues [21] using BLAST. CircRNA sequences on the same chromosomes with more than 90% similarity were considered identical.

The R-Bioconductor package Noiseq (v2.28.0) [70] was used to quantify the expression level and to detect the differential expression pattern of circRNAs between samples. Trimmed Mean of M values (TMM) was used as the normalization method. A circRNA was considered differentially expressed if its q value was ≥ 0.8. Plots were generated using the R software (v4.0.4) [71] with ggplot2 (v3.3.3) [72], VennDiagram (v1.7.1) [73], and gplots (v3.1.1) [74] libraries.

### Analysis of circRNA–miRNA interactions

To analyze the circRNA-miRNA interactions, we first downloaded the FASTA sequence of known miRNAs for soybean from miRbase database [75], and then we predicted the potential interactions between miRNAs and list of our identified differential expressed circRNAs using Targetfinder tool (v1.7) (https://github.com/carringtonlab/TargetFinder). We also predicted the mRNA targets of those miRNAs that had binding sites on circRNAs using Targetfinder. Cytoscape software (v3.8.2) [76] was used to visualize the circRNA–miRNA interaction network.

### Functional enrichment analysis

Gene Ontology (GO) enrichment analysis of the host genes of differentially expressed circRNAs, and also the miRNA targets was carried out using topGO package (v2.42.0) [77] with classic algorithm and fisher’s exact test. Then, we calculated the corrected *p*-value based on the Benjamini–Hochberg multiple testing correction (FDR < 0.05) to identify significant GO terms. To summarize the significant GO terms, we used ClueGO (v2.5.8) [78] with its “preselected Functions”.

### Circular RNA validation

We used RNase R treatment and PCR with divergent primers followed by Sanger sequencing to validate some of our identified circRNAs. Briefly, poly(A) was added to 10 µg of total RNA from each treatment using the Poly(A) Tailing Kit (Thermo Fisher Scientific AM1350) following the manufacturer’s protocol. Then, RNA samples were cleaned up using ethanol precipitation method [79] and dissolved in nuclease-free water. The RNAs then were subjected to RNase R (Part number: E0111-20D1, Lucigen) treatment following the manufacturer’s instruction in a 20 µl reaction in the presence of lithium chloride containing buffer [80]. The RNA samples were cleaned up using ethanol precipitation protocol and reverse transcribed into complementary DNA (cDNA) using SuperScript™ III Reverse Transcriptase (Invitrogen, Carlsbad, CA, USA) in the presence of random hexamers. The sequence of 30 identified circRNAs that were predicted to be involved in SAM development were used for designing divergent primers [81] using PerlPrimer [82]. The PCR cycles were as follows: initial step at 95 °C for 5 min; followed by 40 cycles at 95 °C for 30 S, optimized annealing temperature (Additional file 1: Table S4) for 40 s, and 72 °C for 30 s; and then a final cycle at 72 °C for 5 min. Electrophoresis of agarose gel (1%) was used to visualize the PCR products, and Wizard® SV Gel and PCR Clean-Up System (Promega, Madison, WI, USA) was used to recover the desired bands from the agarose gel. The recovered amplicons were cloned into the pJET1.2/blunt vector using the CloneJET PCR Cloning Kit (Thermo Scientific, Vilnius, Lithuania) and subjected to Sanger sequencing to validate the back-spliced junction sites.

## Supplementary Information

**Additional file 1: Table S1.** Summary statistics of RNA sequencing data. **Table S2.** CircRNAs found using four detection algorithms in all samples and their biological replicates. **Table S3.** List of 384 unique identified circRNAs with their annotation. **Table S4.** List of divergent primers used for experimental validation of selected circRNAs.

**Additional file 2: Table S5.** BlAST result for identification of reverse complementary sequences in upstream and downstream introns of circRNAs and control exons.

**Additional file 3: Table S6.** BlAST results for identification of reverse complementary sequences in 2000 bp upstream and downstream sequence of circRNAs and control exons.

**Additional file 4: Table S7.** The expression profile of circRNAs in response to short-day photoperiod treatment. **Table S8.** Predicted miRNA-circRNA interactions. **Table S9.** GO annotation of circRNA parental genes. **Table S10.** predicted miRNAs’ target genes. **Table S11.** GO annotation of miRNA target genes. **Table S12.** List of control exons used for comparison analysis.

**Additional file 5: Note S1.** Supporting information for the experimental validation of circRNAs using divergent primers and Sanger sequencing.

## Data Availability

All data generated or analysed during this study are included in this published article and its supplementary information files. The datasets generated and analysed during the current study are available in the National Center for Biotechnology Information (NCBI) repository under accession number PRJNA893233. Temporary access to the RNA sequencing data at: https://dataview.ncbi.nlm.nih.gov/object/PRJNA893233?reviewer=sdo2rqhs0iuta1umsdu685r1m4.

## Abbreviations

AP1: APETALA1
CAL: CAULIFLOWER
CO: CONSTANS
FT: FLOWERING LOCUS T
FUL: FRUITFULL
GO: Gene Ontology
LD: Long Day
MFE: Minimum Free Energy
PP2C: PROTEIN PHOSPHATASE TYPE 2C
SAM: Shoot Apical Meristem
SD: Short Day
SOC1: SUPPRESSOR OF OVEREXPRESSION OF CONSTANS 1
SnRK2: SUCROSE-NON-FERMENTING-1-RELATED PROTEIN KINASE-2S
ZDS: ZETA-CAROTENE DESATURASE
