# Supplementary material for: Circular RNAs modulate the floral fate acquisition in soybean shoot apical meristem
Source: BMC Plant Biol. 2023 Jun 16;23:322. doi: 10.1186/s12870-023-04319-3 (PMC10273651; doi:10.1186/s12870-023-04319-3)
Supplement: Supplementary file 5 — Additional file 5: Note S1. Supporting information for the experimental validation of circRNAs using divergent primers and Sanger sequencing. [file 12870_2023_4319_MOESM5_ESM.pdf]

Note S1: Supporting information for the experimental validation of circRNAs using divergent primers and Sanger sequencing

Gene ID: Glyma.13G151100.Wm82.a4.v1 ➔ 16 exons

CircRNA ID: Gm13:25614859-25616046 ➔ exons 2 – 5

Exon 2:

GTGAATGGCTGCCTATCATACTCTGACAAAATTCCAGATGGCTTTTACCTGATTCATGGGATGAATTCCTTTGTCTGGACCTTGTGCACTGATCT
GCATGAAAATGGCCGAATTCCATCAGTTGATATGCTGAAGTCTGTGAATCCCTGCGTCGTTTCTTCACTTGAAGTAGTTAT[R:GGTGGATCGAC
GCAGTGACCCCA]GCTTAAGAGATCTGCAAAATAATGTTCATAACATTTCTTGTACTAGCATAACAACAACAGATGTTGTAGATAAACTTTCCA
AGCTGGTTTGCAACCGTATGGG

Exon 5:

GGAGTATCTTGTTGATTTAATTGGAAAGCCAGGAAACTTAT[F:CTGAGCCTGATTCCTTGCTCAATGGTCC]ATCTTCCATCTCATTTTCTTCACC
CTTGCGCTTTCCACGACTTAAACCGGCTGAACCTACCATTGATTTCAGGTCATTGGCCAAACAGTATTTCTCGGATTGTGTGTCTCTTGAGCTTG
TCTTCGACAACAACTCTGCAG

CircRNA junction:

[F:CTGAGCCTGATTCCTTGCTCAATGGTCC]ATCTTCCATCTCATTTTCTTCACCCTTGCGCTTTCCACGACTTAAACCGGCTGAACCTACCATTG
ATTTCAGGTCATTGGCCAAACAGTATTTCTCGGATTGTGTGTCTCTTGAGCTTGTCTTCGACAACAACTCTGCAGGTGAATGGCTGCCTATCATA
CTCTGACAAAATTCCAGATGGCTTTTACCTGATTCATGGGATGAATTCCTTTGTCTGGACCTTGTGCACTGATCTGCATGAAAATGGCCGAATT
CCATCAGTTGATATGCTGAAGTCTGTGAATCCCTGCGTCGTTTCTTCACTTGAAGTAGTTAT[R:GGTGGATCGACGCAGTGACCCCA]

Gel electrophoresis and Sanger Sequencing:

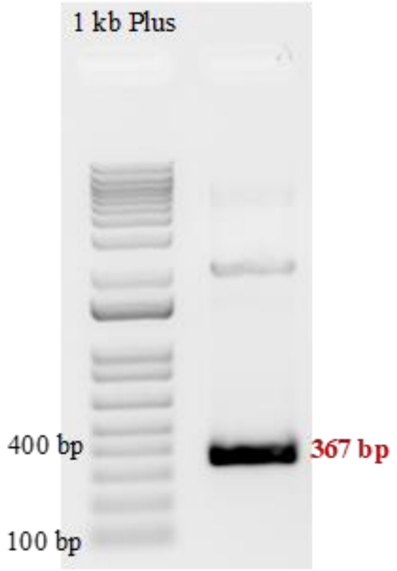

CCCGAAATGTAGGAGNCTTCTAGAAAGAT[F:CTGAGCCTGATTCCTTGCTCAATGGTCC]ATC
TTCCATCTCATTTTCTTCACCCTTGCGCTTTCCACGACTTAAACCGGCTGAACCTACCATTGAT
TTCAGGTCATTGGCCAAACAGTATTTCTCGGATTGTGTGTCTCTTGAGCTTGTCTTCGACAAC
AACTCTGCAGGTGAATGGCTGCCTATCATACTCTGACAAAATTCCAGATGGCTTTTACCTGAT
TCATGGGATGAATTCCTTTGTCTGGACCTTGTGCACTGATCTGCATGAAAATGGCCGAATTCC
ATCAGTTGATATGCTGAAGTCTGTGAATCCCTGCGTCGTTTCTTCACTTGAAGTAGTTAT[R:G
GTGGATCGACGCAGTGACCCCA]ATCTTGCTGAAAAACTCGAGCCATCCGGAAGATCTGGCG
GCCGCTCTCCCTATAGTGAGTCGTATTACGCCGGATGGATATGGTGTTCAGGCACAAGTGTTA
AAGCAGTTGATTTTATTCACTATGATGAAAAAAACAATGAATGGAACCTGCTCCAAGTTAAA
AATAGAGATAATACCGAAAACTCATCGAGTAGTAAGATTAGAGATAATACAACAATAAAAA
AATGGTTTAGAACTTACTCACAGCGTGATGCTACTAATTGGGACAATTTTCCAGATGAAGTAT
CATCTAAGAATTTAAATGAAGAAGACTTCAGAGCTTTTGTTAAAAATTATTTGGCAAAAATA
ATATAATTCGGCTGCAGGGGCGGCCTCGTGATACGCCTATTTTTATAGGTTAATGTCATGATA
ATAATGGTTTCTTAGACGTCAGGTGGCACTTTTCGGGGAAATGTGCGCGGAACCCCTATTTGT
TTATTTTTCTAAATACATTCAAATATGTATCCGCTCATGAGACAATAACCCTGATAAATGCTT
CAATAATATTGAAAAAGGAAGAGTATGAGTATTCAACATTTCCGTGTCGCCCTTATTCCCTTT
TTTGCGGCATTTTGCCTTCCTGTTTTTGCTCACCCAGAAACGCTGGTGAAAGTAAAAGATGCT
GAAGATCAGTTGGGTGCACGAGTGGGTTACATCGAACTGGATCTCAACAGCGGTAAGATCCT
TGAGAGTTTCGCCCCAAGAACGTTTTCCATGATGAGCACTTTTAAAGTTCTGCTAGNGGGCCG
GTATTATCCCGAATGACGCCGGGCAAGAACAACTCGGTCGCGCCAAACCTATTCCAGATGGA
CTGGTTGANNACNCCCCNNTCCNNAAAAANCCTNACGGAGGGTGAAAAGAAAAAAATTTGG
GGGGGGCCCCACCCGGGGGAAAAACCGGGGCCCCTTTTTTTTAAAAAAAAGGGGAAAAAAA
AAAAACCCCCTTTTTTACAAGGGGGGANTTAACCCCCNNTTTTGGGGAAACAGAAAAAAAA
AACCCACAACNNNNNAAGGCCCCCCACGGGCGAAANNNAAACNNATGCCCANNAAATNAG
AAACTTCTTCCTCCCCCCCCTTAATGGGGGGGGGG

Gene ID: Glyma.04G192300.Wm82.a4.v1 ➔ 44 exons

CircRNA ID: Gm04:45203808-45204445 ➔ exons 4 – 7

Exon 4:

TCTTGACGAAATTCATGATT[R:GCAAATGATGGCCCAATGCTAAGT]ATTGTTTTGAAAGTATTCAAGTTTAGATGTCCATGATTGAAAGCAAT
GATTGTCAAAGC

Exon 6:

CCTTTTTGTTCTTTTTG[F:ACGGTTTAGGTTTCAGCAAGAACGG]TGAATCTGGCCTCATTGGCCAGTGTAACTCAAAACAAGCAGGCGAC

Exon 7:

CTGCATATAATTTCAAATTAGAATAAGTCCACCAACAAGCCAAAATTTCATATGGCACCAGTCCTGCTAACCAAAAGTATTCGTTGAAGTCATC
ATAATTCCTCCATGCTGAATGTGCAGCTTTCCCATTGTTGTTTCTACCAGCTTC

CircRNA junction:

[F:ACGGTTTAGGTTTCAGCAAGAACGG]TGAATCTGGCCTCATTGGCCAGTGTAACTCAAAACAAGCAGGCGACCTGCATATAATTTCAAATTA
GAATAAGTCCACCAACAAGCCAAAATTTCATATGGCACCAGTCCTGCTAACCAAAAGTATTCGTTGAAGTCATCATAATTCCTCCATGCTGAAT
GTGCAGCTTTCCCATTGTTGTTTCTACCAGCTTCTCTTGACGAAATTCATGATT[R:GCAAATGATGGCCCAATGCTAAGT]

Gel electrophoresis and Sanger Sequencing:

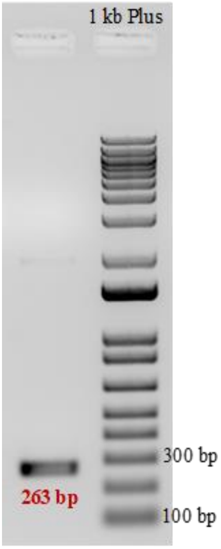

CCCGAAATGTAGGAGATCTTCTAGAAAGAT[F:ACGGTTTAGGTTTCAGCAAGAACGG]TGAATCTGG
CCTCATTGGCCAGTGTAACTCAAAACAAGCAGGCGACCTGCATATAATTTCAAATTAGAATAAGTC
CACCAACAAGCCAAAATTTCATATGGCACCAGTCCTGCTAACCAAAAGTATTCGTTGAAGTCATCA
TAATTCCTCCATGCTGAATGTGCAGCTTTCCCATTGCTGTTTCTACCAGCTTCTCTTGACGAAATTCA
TGATT[R:GCAAATGATGGCCCAATGCTAAGT]ATCTTGCTGAAAAACTCGAGCCATCCGGAAGATCT
GGCGGCCGCTCTCCCTATAGTGAGTCGTATTACGCCGGATGGATATGGTGTTCAGGCACAAGTGTT
AAAGCAGTTGATTTTATTCACTATGATGAAAAAAACAATGAATGGAACCTGCTCCAAGTTAAAAAT
AGAGATAATACCGAAAACTCATCGAGTAGTAAGATTAGAGATAATACAACAATAAAAAAATGGTT
TAGAACTTACTCACAGCGTGATGCTACTAATTGGGACAATTTTCCAGATGAAGTATCATCTAAGAA
TTTAAATGAAGAAGACTTCAGAGCTTTTGTTAAAAATTATTTGGCAAAAATAATATAATTCGGCTGC
AGGGGCGGCCTCGTGATACGCCTATTTTTATAGGTTAATGTCATGATAATAATGGTTTCTTAGACGT
CAGGTGGCACTTTTCGGGGAAATGTGCGCGGAACCCCTATTTGTTTATTTTTCTAAATACATTCAAA
TATGTATCCGCTCATGAGACAATAACCCTGATAAATGCTTCAATAATATTGAAAAAGGAAGAGTAT
GAGTATTCAACATTTCCGTGTCGCCCTTATTCCCTTTTTTGCGGCATTTTGCCTTCCTGTTTTTGCTCA
CCCAGAAACGCTGGTGAAAGTAAAAGATGCTGAAGATCAGTTGGGTGCACGAGTGGGTTACATCG
AACTGGATCTCAACAGCGGTAAGATCCTTGAGAGTTTTCGCCCCGAAGAACGTTTTCCAATGATGA
GCACTTTTAAAGTTCTGCTATGTGGCGCGGTATTATCCCGTATTGACGCCGGGCAAGAGCAACTCG
GTCGCCGCATACCCTATTCTCAGAATGACTTGGTTGAGTACTCACCAGTCCNAGAAAAGCATCTTAC
GGATGGCATGACAGTAAGAGAATTATGCAGGGCTGCCNTAACCATGAGTGATAANCTGGCGGCCA
ATTTANNTTTGGA

Gene ID: Glyma.04G192300.Wm82.a4.v1 ➔ 12 exons

CircRNA ID: Gm04:45203808-45204445 ➔ exons 7 – 10

Exon 7:

CATTGCAGTAGCATTAGAAAAGAAATCAGCATATGG[R:CCTTGGTGTTATGACCCATCTAGAGAGAG]AACCATTTCCAAGAGAAGAAAAATT
CATTAAGTGAAC

Exon 9:

TAGCTGTAAATTATACTGTGATGGAGATGG[F:AGCAACAGAGGGCTTAAATGCTTTGG]TTAAATTTGTTCCTTCAGATTTAGGAGGCATCAAG
GGTGGTGGGGCATTTGAGACATGTAGTCTTTCCCATAACTCAGAACAATTAGTTTTCCAGAAACCAATTTTTGAATGCTCACGATCATACATTA
CAAGAGTGTTACGAACAACAATGC

Exon 10:

CTCCTAAAAGAGTAGTTGGATCATTGCCATTTGAGAAAACCCCTAGACAATACGCACCTCGCACTTTTGAATG

CircRNA junction:

[F:AGCAACAGAGGGCTTAAATGCTTTGG]TTAAATTTGTTCCTTCAGATTTAGGAGGCATCAAGGGTGGTGGGGCATTTGAGACATGTAGTCTT
TCCCATAACTCAGAACAATTAGTTTTCCAGAAACCAATTTTTGAATGCTCACGATCATACATTACAAGAGTGTTACGAACAACAATGCCTCCTA
AAAGAGTAGTTGGATCATTGCCATTTGAGAAAACCCCTAGACAATACGCACCTCGCACTTTTGAATGCATTGCAGTAGCATTAGAAAAGAAAT
CAGCATATGG[R:CCTTGGTGTTATGACCCATCTAGAGAGAG]

Gel electrophoresis and Sanger Sequencing:

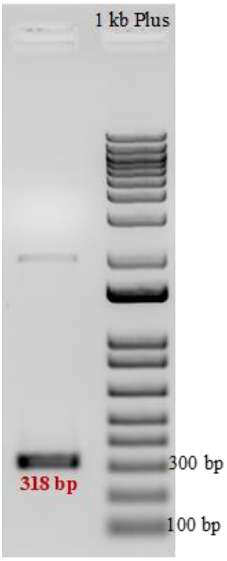

CCCGGANTCGGAATGGCTCGAGTTTTTCAGCAAGAT[F:AGCAACAGAGGGCTTAAATGCTTTGG]TTAAATT
TGTTCCTTCAGATTTAGGAGGCATCAAGGGTGGTGGGGCATTTGAGACATGTAGTCTTTCCCATAACTCAGA
ACAATTAGTTTTCCAGAAACCAATTTTTGAATGCTCACGATCATACATTACAAGAGTGTTACGAACAACAAT
GCCTCCTAAAAGAGTAGTTGGATCATTGCCATTTGAGAAAACCCCTAGACAATACGCACCTCGCACTTTTGA
ATGCATTGCAGTAGCATTAGAAAAGAAATCAGCATATGG[R:CCTTGGTGTTATGACCCATCTAGAGAGAG]A
TCTTTCTAGAAGATCTCCTACAATATTCTCAGCTGCCATGGAAAATCGATGTTCTTCTTTTATTCTCTCAAGA
TTTTCAGGCTGTATATTAAAACTTATATTAAGAACTATGCTAACCACCTCATCAGGAACCGTTGTAGGTGGC
GTGGGTTTTCTTGGCAATCGACTCTCATGAAAACTACGAGCTAAATATTCAATATGTTCCTCTTGACCAACTT
TATTCTGCATTTTTTTTGAACGAGGTTTAGAGCAAGCTTCAGGAAACTGAGACAGGAATTTTATTAAAAATT
TAAATTTTGAAGAAAGTTCAGGGTTAATAGCATCCATTTTTTGCTTTGCAAGTTCCTCAGCATTCTTAACAAA
AGACGTCTCTTTTGACATGTTTAAAGTTTAAACCTCCTGTGTGAAATTATTATCCGCTCATAATTCCACACAT
TATACGAGCCGGAAGCATAAAGTGTAAAGCCTGGGGTGCCTAATGAGTGAGCTAACTCACATTAATTGCGT
TGCGCTCACTGCCAATTGCTTTCCAGTCGGGAAACCTGTCGTGCCAGCTGCATTAATGAATCGGCCAACGCG
CGGGGAGAGGCGGTTTGCGTATTGGGCGCTCTTCCGCTTCCTCGCTCACTGACTCGCTGCGCTCGGTCGTTC
GGCTGCGGCGAGCGGTATCAGCTCACTCAAAGGCGGTAATACGGTTATCCACAGAATCAGGGGATAACGCA
GGAAAGAACATGTGAGCAAAAGGCCAGCAAAAGGCCAGGAACCGTAAAAAGGCCCCGTTGCTGGCGTTTT
TCCATAGGCTCCCCCCCCTGACAAGCATCNAAAAATCGACGCTCAAGTCAAAGGTGGCGAAACCCGACGGN
ACTATAAGAAACCAGGCGTTTCCCCCTGGAAGCTCCCCGGGGGCTCCCCGTTCCNNCCNGCCCCTTAACGAG
ANNCGGGTCCCCCTTTCCCCCNGGGAAAGGGGGCTTTTTCCACTCCCGGGGAAATTTCCATTGGGGGAGGG
GCT

Gene ID: Glyma.04G000400.Wm82.a4.v1 ➔ 18 exons

CircRNA ID: Gm04:60681-61366 ➔ exons 9 and 10

Exon 9:

CTTCAAGGATGATTACACGGCTGAAGTTATGCTT[R:GTAGAACAGTTGATCTAGAGCCCACT]TGTAATGAC

Exon 10:

TGCAATTT[F:TGTAGTAAGCAGTTAACTCTCCTGGC]CGTTCAGTTTGAACTGGTTCAAAATCCAAGTG

CircRNA junction:

[F:TGTAGTAAGCAGTTAACTCTCCTGGC]CGTTCAGTTTGAACTGGTTCAAAATCCAAGTGCTTCAAGGATGATTACACGGCTGAAGTTATGCTT
[R:GTAGAACAGTTGATCTAGAGCCCACT]

Gel electrophoresis and Sanger Sequencing:

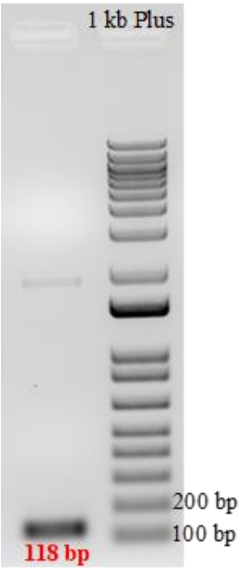

CCCGAATTCGGATGGCTCGAGTTTTNAGCAAGAT[F:TGTAGTAAGCAGTTAACTCTCCTGGC]CGT
TCAGTTTGAACTGGTTCAAAATCCAAGTGCTTCAAGAATGATCACACGGCTGAAGTTATGCTT[R:
GTAGAACAGTTGATCTAGAGCCCACT]ATCTTTCTAGAAGATCTCCTACAATATTCTCAGCTGCCA
TGGAAAATCGATGTTCTTCTTTTATTCTCTCAAGATTTTCAGGCTGTATATTAAAACTTATATTAA
GAACTATGCTAACCACCTCATCAGGAACCGTTGTAGGTGGCGTGGGTTTTCTTGGCAATCGACTC
TCATGAAAACTACGAGCTAAATATTCAATATGTTCCTCTTGACCAACTTTATTCTGCATTTTTTTT
GAACGAGGTTTAGAGCAAGCTTCAGGAAACTGAGACAGGAATTTTATTAAAAATTTAAATTTTG
AAGAAAGTTCAGGGTTAATAGCATCCATTTTTTGCTTTGCAAGTTCCTCAGCATTCTTAACAAAA
GACGTCTCTTTTGACATGTTTAAAGTTTAAACCTCCTGTGTGAAATTATTATCCGCTCATAATTCC
ACACATTATACGAGCCGGAAGCATAAAGTGTAAAGCCTGGGGTGCCTAATGAGTGAGCTAACTC
ACATTAATTGCGTTGCGCTCACTGCCAATTGCTTTCCAGTCGGGAAACCTGTCGTGCCAGCTGCA
TTAATGAATCGGCCAACGCGCGGGGAGAGGCGGTTTGCGTATTGGGCGCTCTTCCGCTTCCTCGC
TCACTGACTCGCTGCGCTCGGTCGTTCGGCTGCGGCGAGCGGTATCAGCTCACTCAAAGGCGGTA
ATACGGTTATCCACAGAATCAGGGGATAACGCAGGAAAGAACATGTGAGCAAAAGGCCAGCAA
AAGGCCAGGAACCGTAAAAAGGCCGCGTTGCTGGCGTTTTTCCATAGGCTCCGCCCCCCTGACG
AGCATCACAAAAATCGACGCTCAAGTCAGAGGTGGCGAAACCCGACAGGACTATAAAGATACC
AGGCGTTTCCCCCTGGAAGCTCCCTCGTGCGCTCTCCTGTTCCGACCCTGCCGCTTACCGGATACC
TGTCCGCCTTTCTCCCTTCGGGAAGCGTGGCGCTTTCTCATAGCTCACGCTGTAGGTATCTCAGTT
CGGTGTAGGTCGTTCGCTCCAAGCTGGGCTGGGTGCCCAAACCCCCGTTCAGCCGAACGCTGGG
CCTTATCCGGTAACTATCGTCTTGAGTCCAACCCGGGAANAACCCGAATTTTCGCCCCTGGCACC
ACCCCCGGGTANANGATTAACAAAAAGAAGGNTTTTGGGGGGGCCCAAAAATTTTTTTT

Gene ID: Glyma.19G202400.Wm82.a4.v1 ➔ 15 exons

CircRNA ID: Gm19:46460019-46460732 ➔ exons 2 and 3

Exon 2:

ATCAATCTCAATGGCGTGTTTTGATTGGTAAAGTACGCGCGACGGAACGAGGCATGGCAGAAGTGGCTAAACCGAGTTCTGTGCCTCCGA[R:GCTCTCATCGAATTGGTTCAGGACCT]TTGAAAAGTGGATCTTTGGTTAGAAAGAAGACACCGTCAGAACTTAGG

Exon 3:

GGAGAGT[F:TGTTGAAGCGGGCAAGTAATGTGGAC]CTTACTGATGAATCTCAATCCACCTTGGCTGATCCTACGAA

CircRNA junction:

[F:TGTTGAAGCGGGCAAGTAATGTGGAC]CTTACTGATGAATCTCAATCCACCTTGGCTGATCCTACGAAATCAATCTCAATGGCGTGTTTTGATTGGTAAAGTACGCGCGACGGAACGAGGCATGGCAGAAGTGGCTAAACCGAGTTCTGTGCCTCCGA[R:GCTCTCATCGAATTGGTTCAGGACCT]

Gel electrophoresis and Sanger Sequencing:

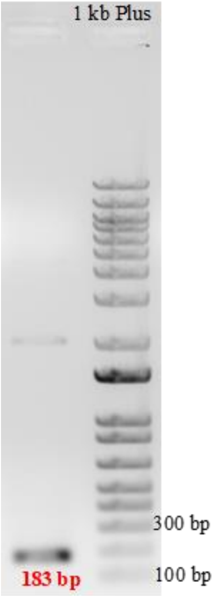

CCCGGNTTCGGATGGCTCGAGTTTTTCAGCAAGAT[F:TGTTGAAGCGGGCAAGTAATGTGGAC]CTTACT
GATGAATCTCAATCCACCTTGGCTGATCCTACGAAATCAATCTCAATGGCGTGTTTTGATTGGTAAAGTA
CGCGCGACGGAACGAGGCATGGCAGAAGTGGCTAAACCGAGTTCTGTGCCTCCGA[R:GCTCTCATCGAA
TTGGTTCAGGACCT]ATCTTTCTAGAAGATCTCCTACAATATTCTCAGCTGCCATGGAAAATCGATGTTCT
TCTTTTATTCTCTCAAGATTTTCAGGCTGTATATTAAAACTTATATTAAGAACTATGCTAACCACCTCATC
AGGAACCGTTGTAGGTGGCGTGGGTTTTCTTGGCAATCGACTCTCATGAAAACTACGAGCTAAATATTC
AATATGTTCCTCTTGACCAACTTTATTCTGCATTTTTTTTGAACGAGGTTTAGAGCAAGCTTCAGGAAAC
TGAGACAGGAATTTTATTAAAAATTTAAATTTTGAAGAAAGTTCAGGGTTAATAGCATCCATTTTTTGCT
TTGCAAGTTCCTCAGCATTCTTAACAAAAGACGTCTCTTTTGACATGTTTAAAGTTTAAACCTCCTGTGT
GAAATTATTATCCGCTCATAATTCCACACATTATACGAGCCGGAAGCATAAAGTGTAAAGCCTGGGGTG
CCTAATGAGTGAGCTAACTCACATTAATTGCGTTGCGCTCACTGCCAATTGCTTTCCAGTCGGGAAACCT
GTCGTGCCAGCTGCATTAATGAATCGGCCAACGCGCGGGGAGAGGCGGTTTGCGTATTGGGCGCTCTTC
CGCTTCCTCGCTCACTGACTCGCTGCGCTCGGTCGTTCGGCTGCGGCGAGCGGTATCAGCTCACTCAAAG
GCGGTAATACGGTTATCCACAGAATCAGGGGATAACGCAGGAAAGAACATGTGAGCAAAAGGCCAGCA
AAAGGCCAGGAACCGTAAAAAGGCCGCGTTGCTGGCGTTTTTCCATAGGCTCCGCCCCCTGACGAGCAT
CACAAAAATCGACGCTCAAGTCAGAGGTGGCGAAACCCGACAGGACTATAAAGATACCAGGCGTTTCC
CCCTGGAAGCTCCCTCGTGCGCTCTCCTGTTCGGACCNGGCGCTTACGGAAACCTGTCGGCTTNCCCCTT
CGGGAACGTGGGGCTTTCCCAAAACTCCACCGGAAGGNTCNCAATTCCGGGTAAGTCCTTCCCCCAACT
GGGGTGGGGG

Gene ID: Glyma.17G262300.Wm82.a4.v1 ➔ 5 exons

CircRNA ID: Gm17:41684169-41684774 ➔ exons 2 - 4

Exon 2:

AGATAGCAAATCCAGTCCAAGCTCATCCAAACTTGGAACAGCCGTTGACAGACTTTGAGGGTTCCATTGAGGGTATTCATGCCAGTT[R:CATTA
GTTTACTCACGCCTGGCCACA]CGTCTTCGTTGGGAGTACCCAATAGC

Exon 4:

CAAATATGCAGCCTACGGACCAAATGTCCACCGCCATTGAGTAATGG[F:GTAGCACCCAAGAGGACTTCAGGAGC]TCTATACCACAGGGTTA
GTATCTCATGTGTATATTTCTTAATCGGCACAGTAAATGCTCGAGCGAGTCCAAGATCAGCAATTTTAAGCATCATGGTTTTTGGGTCCATCAA
GAGATTGTGAGGTTTCAAGTCC

CircRNA junction:

[F:GTAGCACCCAAGAGGACTTCAGGAGC]TCTATACCACAGGGTTAGTATCTCATGTGTATATTTCTTAATCGGCACAGTAAATGCTCGAGCGA
GTCCAAGATCAGCAATTTTAAGCATCATGGTTTTTGGGTCCATCAAGAGATTGTGAGGTTTCAAGTCCAGATAGCAAATCCAGTCCAAGCTCAT
CCAAACTTGGAACAGCCGTTGACAGACTTTGAGGGTTCCATTGAGGGTATTCATGCCAGTT[R:CATTAGTTTACTCACGCCTGGCCACA]

Gel electrophoresis and Sanger Sequencing:

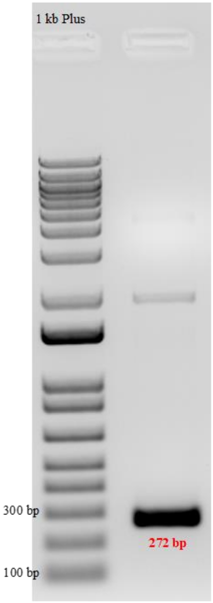

CCCGAAATGTAGGAGATCTTCTAGAAAGAT[F:GTAGCACCCAAGAGGACTTCAGGAGC]TCTAT
ACCACAGGGTTAGTATCTCATGTGTATATTTCTTAATCGGCACAGTAAATGCTCGAGCGAGTCC
AAGATCAGCAATTTTAAGCATCATGGTTTTTGGGTCCATCAAGAGATTGTGAGGTTTCAAGTCC
AGATAGCAAATCCAGTCCAAGCTCATCCAAACTTGGAACAGCCGTTGACAGACTTTGAGGGTT
CCATTGAGGGTATTCATGCCAGTT[R:CATTAGTTTACTCACGCCTGGCCACA]ATCTTGCTGAAA
AACTCGAGCCATCCGGAAGATCTGGCGGCCGCTCTCCCTATAGTGAGTCGTATTACGCCGGATG
GATATGGTGTTCAGGCACAAGTGTTAAAGCAGTTGATTTTATTCACTATGATGAAAAAAACAAT
GAATGGAACCTGCTCCAAGTTAAAAATAGAGATAATACCGAAAACTCATCGAGTAGTAAGATT
AGAGATAATACAACAATAAAAAAATGGTTTAGAACTTACTCACAGCGTGATGCTACTAATTGG
GACAATTTTCCAGATGAAGTATCATCTAAGAATTTAAATGAAGAAGACTTCAGAGCTTTTGTTA
AAAATTATTTGGCAAAAATAATATAATTCGGCTGCAGGGGCGGCCTCGTGATACGCCTATTTTT
ATAGGTTAATGTCATGATAATAATGGTTTCTTAGACGTCAGGTGGCACTTTTCGGGGAAATGTG
CGCGGAACCCCTATTTGTTTATTTTTCTAAATACATTCAAATATGTATCCGCTCATGAGACAATA
ACCCTGATAAATGCTTCAATAATATTGAAAAAGGAAGAGTATGAGTATTCAACATTTCCGTGTC
GCCCTTATTCCCTTTTTTGCGGCATTTTGCCTTCCTGTTTTTGCTCACCCAGAAACGCTGGTGAA
AGTAAAAGATGCTGAAGATCAGTTGGGTGCACGAGTGGGTTACATCGAACTGGATCTCAACAG
CGGTAAGATCCTTGAGAGTTTTCGCCCCGAAGAACGTTTTCCAATGATGAGCACTTTTAAAGTT
CTGCTATGTGGCNCGGTATTATCCCGTATTGACGCCGGGCAAGAGCAACTCGGTCGCCGCAAN
CCCTATTTTCAAATGACTTGGTTGAATACTCACAGTCCAAGAAAGCATCTTACGGATGGCTGGA
NGGTAGAGAATNTGCAGGCTGCCTAACCTGAAGGAAAACCTGGGGCCAATTACTTTTAAAAAA
ATTGNGAACAANGNTAACCCTTTTTTTCCCAAAGGGGNNNATTTNNTCCCCCTTTTTTGGGGAA
CCAGGTGTAAANACCCCCAAAAAAA

Gene ID: Glyma.05G075100.Wm82.a4.v1 ➔ 7 exons

CircRNA ID: Gm05:9376287-9377127 ➔ exons 3 - 5

Exon 3:

GGAATAACTGCAGCATCATTTATCTCTGAATGAGATAGGAGTAACTCTTCTAGCTCTGCCGGAGCAAC

Exon 4:

CTGGTAGCCCTTGTATTTAATCAACTCTTTCAACCTATCTACAACATACAAGAAACCTTTGCTATCAAAATAA[R:CAGAGGTCCCCAGTCCTTA
ACCACC]CATCCACCAAAGTTGCTGAAGTTGCTTTTGGGTCACCAGAATAAC

Exon 5:

CTTTCATAACATAAGGTCCTCTGATCCAGAGTTCTCCTTGTTCACCGGGAAACAT[F:GGCTTCCCCTGTTTCTGGGTTCAC]AATTTTGGCTTCTA
TATTTGGTATCAGTTTACCTGTTGCTCCCACCTGATTTGCCTCCTCTGGAGTGGTTCGGGTAACTGCAGACTCAGTTAAACCGTATCC

CircRNA junction:

[F:GGCTTCCCCTGTTTCTGGGTTCAC]AATTTTGGCTTCTATATTTGGTATCAGTTTACCTGTTGCTCCCACCTGATTTGCCTCCTCTGGAGTGGTT
CGGGTAACTGCAGACTCAGTTAAACCGTATCCGGAATAACTGCAGCATCATTTATCTCTGAATGAGATAGGAGTAACTCTTCTAGCTCTGCCGG
AGCAACCTGGTAGCCCTTGTATTTAATCAACTCTTTCAACCTATCTACAACATACAAGAAACCTTTGCTATCAAAATAA[R:CAGAGGTCCCCAG
TCCTTAACCACC]

Gel electrophoresis and Sanger Sequencing:

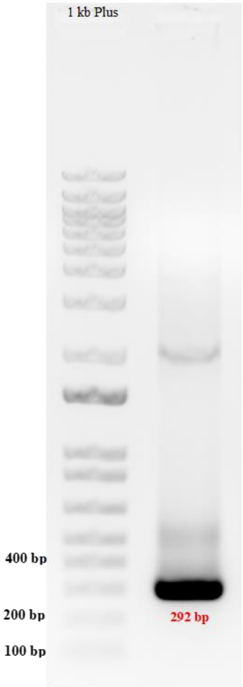

CCCCCAATTCGGATGGCTCGAGTTTTTCAGCAAGAT[F:GGCTTCCCCTGTTTCTGGGTTCAC]AATTTTG
GCTTCTATATTTGGTATCAGTTTACCTGTTGCTCCCACCTGATTTGCCCCCTCTGGAGTGGTTCGGGTA
ACTGCAGACTCAGTTAAACCGTATCCGGAATAACTGCAGCATCATTTATCTCTGAATGAGATAGGAGT
AACTCTTCTAGCTCTGCCGGAGTAACCTGGTAGCCCTTGTATTTAATCAACTCTTTCAACCTATCTACA
ACATACAAGAAACCTTTGCTATCAAAATAA[R:CAGAGGTCCCCAGTCCTTAACCACC]ATCTTTCTAGA
AGATCTCCTACAATATTCTCAGCTGCCATGGAAAATCGATGTTCTTCTTTTATTCTCTCAAGATTTTCA
GGCTGTATATTAAAACTTATATTAAGAACTATGCTAACCACCTCATCAGGAACCGTTGTAGGTGGCGT
GGGTTTTCTTGGCAATCGACTCTCATGAAAACTACGAGCTAAATATTCAATATGTTCCTCTTGACCAAC
TTTATTCTGCATTTTTTTTGAACGAGGTTTAGAGCAAGCTTCAGGAAACTGAGACAGGAATTTTATTAA
AAATTTAAATTTTGAAGAAAGTTCAGGGTTAATAGCATCCATTTTTTGCTTTGCAAGTTCCTCAGCATT
CTTAACAAAAGACGTCTCTTTTGACATGTTTAAAGTTTAAACCTCCTGTGTGAAATTATTATCCGCTCA
TAATTCCACACATTATACGAGCCGGAAGCATAAAGTGTAAAGCCTGGGGTGCCTAATGAGTGAGCTA
ACTCACATTAATTGCGTTGCGCTCACTGCCAATTGCTTTCCAGTCGGGAAACCTGTCGTGCCAGCTGCA
TTAATGAATCGGCCAACGCGCGGGGAGAGGCGGTTTGCGTATTGGGCGCTCTTCCGCTTCCTCGCTCA
CTGACTCGCTGCGCTCGGTCGTTCGGCTGCGGCGAGCGGTATCAGCTCACTCAAAGGCGGTAATACGG
TTATCCACAGAATCAGGGGATAACGCAGGAAAGAACATGTGAGCAAAAGGCCAGCAAAAGGCCAGG
AACCGTAAAAAGGCCGCGTTGCTGGCGTTTTTCCNTAGGCTCCGCCCCCCTGACGAGCATCCCAAAAT
CGACGCTCAGTCCAAGGGGCCAAACCCGACAGGACTTAAAGAAACCAGGCGTTTCCCCCTGGAAACT
CCCTCGGGGCCTCTCTGTTCCAACCTGGCCCTTACGGAANCTGNCCCCTTTTTCCCCTTTGGAAAGGGG
GGCNTTTCCAACCCCCCCGGAGGGATTTCCCTTGGGGGGGGGGGCTCCCCCCCCCCGGGGGGNNGGG
GGGCAAACCCCCCTNCNCCCNNCCCCGCCCCCTTNTCGGGAATTTCTTTTTTTGGTGCCCCCCCCGGAA
AAANNAAANTNCCCCCNNGGGCNNCCCCCCNTAAANNNANNAAAAAAAAAAGAGNGGTGGGNNNN
NCCACAATTTTNNNGT

Gene ID: Glyma.06G000300.Wm82.a4.v1 ➔ 18 exons

CircRNA ID: Gm06:33087-34718 ➔ exons 9 - 12

Exon 9:

CACTTGGATTTTGAACCAGTTCAAACTGAACGGCCTGGAGAGTTAACTGCTTACTACAAAATTGCAC

Exon 10:

GTCATTACAAGTGGGCTCTAGATCAACT[R:GTTCTACAAGCATAACTTCAGCCGTGTG]ATCATTCTTGAAG

Exon 11:

ATGACATGGAAATAGCACCTGATTTCTTT[F:GATTATTTTGAAGCTGCAGCGACTCTCC]TTGACAAGGATAA

Exon 12:

ATCCATTATGGCTGTTTCCTCATGGAATGACAATGGACAAAAGCAGTTTGTACATGATCCAT

CircRNA junction:

[F:GATTATTTTGAAGCTGCAGCGACTCTCC]TTGACAAGGATAAATCCATTATGGCTGTTTCCTCATGGAATGACAATGGACAAAAGCAGTTTGT
ACATGATCCATCACTTGGATTTTGAACCAGTTCAAACTGAACGGCCTGGAGAGTTAACTGCTTACTACAAAATTGCACGTCATTACAAGTGGG
CTCTAGATCAACT[R:GTTCTACAAGCATAACTTCAGCCGTGTG]

Gel electrophoresis and Sanger Sequencing:

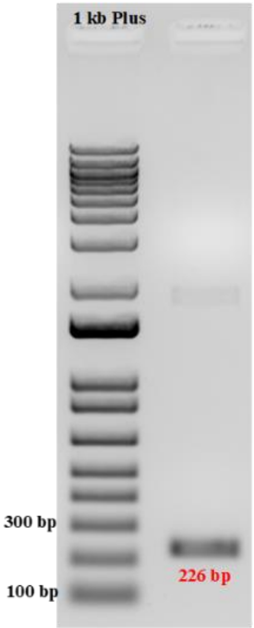

NCCCGAANTCGGAATGGCTCGAGTTTTTCAGCAAGAT[F:GATTATTTTGAAGCTGCAGCGACTCTC
C]TTGACAGGATAAATCCATTATGGCTGTTTCCTCATGGAATGACAATGGACAAAAGCAGTTTGTA
CATGATCCATCACTTGGATTTTGAACCAGTTCAAACTGAACGGCCTGGAGAGTTAACTGCTTACTA
CAAAATTGCACGTCATTACAAGTGGGCTCTAGATCAACT[R:GTTCTACAAGCATAACTTCAGCCGT
GTG]ATCTTTCTAGAAGATCTCCTACAATATTCTCAG[R:CTGCCATGGAAAATCGATGTTCTT]CTTT
TATTCTCTCAAGATTTTCAGGCTGTATATTAAAACTTATATTAAGAACTATGCTAACCACCTCATCA
GGAACCGTTGTAGGTGGCGTGGGTTTTCTTGGCAATCGACTCTCATGAAAACTACGAGCTAAATAT
TCAATATGTTCCTCTTGACCAACTTTATTCTGCATTTTTTTTGAACGAGGTTTAGAGCAAGCTTCAG
GAAACTGAGACAGGAATTTTATTAAAAATTTAAATTTTGAAGAAAGTTCAGGGTTAATAGCATCC
ATTTTTTGCTTTGCAAGTTCCTCAGCATTCTTAACAAAAGACGTCTCTTTTGACATGTTTAAAGTTT
AAACCTCCTGTGTGAAATTATTATCCGCTCATAATTCCACACATTATACGAGCCGGAAGCATAAAG
TGTAAAGCCTGGGGTGCCTAATGAGTGAGCTAACTCACATTAATTGCGTTGCGCTCACTGCCAATT
GCTTTCCAGTCGGGAAACCTGTCGTGCCAGCTGCATTAATGAATCGGCCAACGCGCGGGGAGAGG
CGGTTTGCGTATTGGGCGCTCTTCCGCTTCCTCGCTCACTGACTCGCTGCGCTCGGTCGTTCGGCTG
CGGCGAGCGGTATCAGCTCACTCAAAGGCGGTAATACGGTTATCCACAGAATCAGGGGATAACGC
AGGAAAGAACATGTGAGCAAAAGGCCAGCAAAAGGCCAGGAACCGTAAAAAGGCCGCGTTGCTG
GCGTTTTTCCATAGGCTCCGCCCCCCTGACAAGCATCACAAAAATCGACGCTCAAGTCAGAGGTG
GCGAAACCCGACGGGACTATAAAGATACCAGGCGTTTCCCCCTGGAANCTCCCTCGTGCGCTNCCC
GGTTCCAACCCTGCCGCTTACCGGAAACCTGTCCGCCTTTTTCCCTTCGGGAAGCTNGGGCCTTTC
NCNTAACCTCCNGCGTGAGGTNCTCNTTTCGGGGAAANNCGTTCCNCCCAANNGGGGGGGGGCNN
AACCCCCCTTTTCCCCAACCCGGGCCCTTTCGGGGGTT

Gene ID: Glyma.08G035500.Wm82.a4.v1 ➔ 9 exons

CircRNA ID: Gm08:2820041-2820979 ➔ exons 6 – 8

Exon 6:

GGTGGCATTTTTGTGCTGGAACCTCAGCCATGGAAGTCATATGAAAGCAATCGTAATGTCTCAGAG

Exon 7:

ACCACT[R:GCTGCCAACTACCGTAATATTATGATTCGA]CCAGAACAGTTTCAGGAAATATTACTAGATAAG

Exon 8:

ATTGGATTTCGAACAGTTGAAGATATCACTTCAGGTTT[F:GACAGATAGCAAGACTGGTTTCAACAGG]CCAATTCTGGTTTTTTGCAAATGATC
CGTGCATGCAAATAGTCAG

CircRNA junction:

[F:GACAGATAGCAAGACTGGTTTCAACAGG]CCAATTCTGGTTTTTTGCAAATGATCCGTGCATGCAAATAGTCAGGGTGGCATTTTTGTGCTG
GAACCTCAGCCATGGAAGTCATATGAAAGCAATCGTAATGTCTCAGAGACCACT[R:GCTGCCAACTACCGTAATATTATGATTCGA]

Gel electrophoresis and Sanger Sequencing:

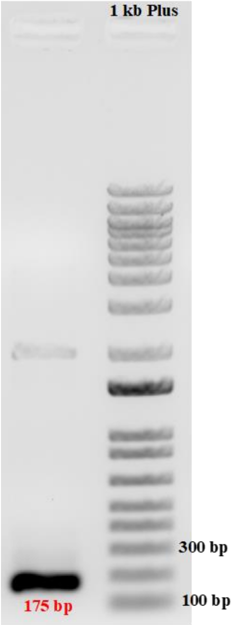

CCCGAANTCGGATGGCTCGAGTTTTTCAGCAAGAT[F:GACAGATAACAAGACTGGTTTCAACAGG]CCAATT
CTGGTTTTTTGCAAATGATCCGTGCATGCAAATAGTCAGGGTGGCATTTTTGTGCTGGAACCTCAGCCATGG
AAGTCATATGAAAGCAATCGTAATGTCTCAGAGACCACT[R:GCTGCCAACTACCGTAATATTATGATTCGA]
ATCTTTCTAGAAGATCTCCTACAATATTCTCAGCTGCCATGGAAAATCGATGTTCTTCTTTTATTCTCTCAAG
ATTTTCAGGCTGTATATTAAAACTTATATTAAGAACTATGCTAACCACCTCATCAGGAACCGTTGTAGGTGG
CGTGGGTTTTCTTGGCAATCGACTCTCATGAAAACTACGAGCTAAATATTCAATATGTTCCTCTTGACCAAC
TTTATTCTGCATTTTTTTTGAACGAGGTTTAGAGCAAGCTTCAGGAAACTGAGACAGGAATTTTATTAAAAA
TTTAAATTTTGAAGAAAGTTCAGGGTTAATAGCATCCATTTTTTGCTTTGCAAGTTCCTCAGCATTCTTAACA
AAAGACGTCTCTTTTGACATGTTTAAAGTTTAAACCTCCTGTGTGAAATTATTATCCGCTCATAATTCCACAC
ATTATACGAGCCGGAAGCATAAAGTGTAAAGCCTGGGGTGCCTAATGAGTGAGCTAACTCACATTAATTGC
GTTGCGCTCACTGCCAATTGCTTTCCAGTCGGGAAACCTGTCGTGCCAGCTGCATTAATGAATCGGCCAACG
CGCGGGGAGAGGCGGTTTGCGTATTGGGCGCTCTTCCGCTTCCTCGCTCACTGACTCGCTGCGCTCGGTCGT
TCGGCTGCGGCGAGCGGTATCAGCTCACTCAAAGGCGGTAATACGGTTATCCACAGAATCAGGGGATAACG
CAGGAAAGAACATGTGAGCAAAAGGCCAGCAAAAGGCCAGGAACCGTAAAAAGGCCGCGTTGCTGGCGTT
TTTCCATAGGCTCCGCCCCCCTGACGAGCATCACAAAAATCGACGCTCAAGTCAGAGGTGGCGAAACCCGA
CAGGACTATAAAGATACCAGGCGTTTCCCCCTGGAAGCTCCCTCGTGCGCTCTCCTGTTCCGACCCTGCCGC
TTACCGGAAACCTGTCCGCCTTTCTCCCTTCGGGAAGCGTGGCGCTTTCCCAAAGCTCACGCTGTAAGGTAT
CCCAGTTCGGGGGAAGGTCGTTNCCTTCCAAGCTGGGGTGGGGGGCCAGAACCCCCCCTATCACGCCCCAC
CCCGGCCCCTTANCGGGAAAATTTTCTTTGTTCCCCCGGGAAAAACAATTTTTCCCCGGNANCCCCCGTAAA
AAAAAAAAAAAGGGTTGGGGGGGGGCCAAAATTTTAGGGGGGGCANCACCCCCCCCCNANAAAAAAATTT
TTTTCCCCC

Gene ID: Glyma.05G001000.Wm82.a4.v1 ➔ 10 exons

CircRNA ID: Gm05:92631-94898 ➔ exons 4 - 8

Exon 4:

CTTATTGTGTAGAACAGATGCTTCTTGATCAAAGAATAGCCATGATTCATCTGGATTTAT

Exon 5:

CTTTGGAGCCAATGTAGCCTTAGAGTCGTCTTGA[R:GGTTGTGCCTTTACTCTTGCTGATCCAT]CAAGTATAGAAGGAAGTTCTCCAATCAGAA
GTTTAGATC

Exon 7:

CTTTATTTGATCATCAACTTGAATGGTTT[F:CAGTAGCAATGACAGGTCCAGCATCG]AGGGCACGGACAGTGAATGCTAATGATACTCCAGTT
TCTTTAACACCATC

Exon 8:

CTGCAATGCCCTTTGAACAGGAGCAGCACCACGATACAATGGCAAAAGGCTAGGGTGAATATTGACTGTTC

CircRNA junction:

[F:CAGTAGCAATGACAGGTCCAGCATCG]AGGGCACGGACAGTGAATGCTAATGATACTCCAGTTTCTTTAACACCATCCTGCAATGCCCTTTG
AACAGGAGCAGCACCACGATACAATGGCAAAAGGCTAGGGTGAATATTGACTGTTCCTTATTGTGTAGAACAGATGCTTCTTGATCAAAGAAT
AGCCATGATTCATCTGGATTTATCTTTGGAGCCAATGTAGCCTTAGAGTCGTCTTGA[R:GGTTGTGCCTTTACTCTTGCTGATCCAT]

Gel electrophoresis and Sanger Sequencing:

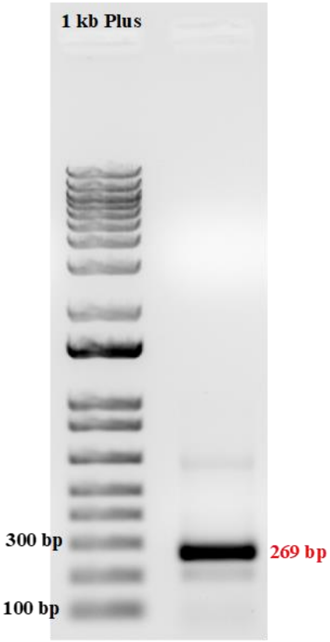

CCCGAAATGTAGGAGNCTTCTAGAAAGAT[F:CAGTAGCAATGACAGGTCCAGCATCG]AGGGCACG
GACAGTGAATGCTAATAATACTCCAGTTTCTTTAACACCATCCTGCAATGCCCTTTGAACAGGAGCA
GCACCACGATACAATGGCAAAAGGCTAGGGTGAATATTGACTGTTCCTTATTGTGTAGAACAGATG
CTTCTTGATCAAAGAATAGCCATGATTCATCTGGATTTATCTTTGGAGCCAATGTAGCCTTAGAGTC
GTCTTGA[R:GGTTGTGCCTTTACTCTTGCTGATCCAT]ATCTTGCTGAAAAACTCGAGCCATCCGGAA
GATCTGGCGGCCGCTCTCCCTATAGTGAGTCGTATTACGCCGGATGGATATGGTGTTCAGGCACAA
GTGTTAAAGCAGTTGATTTTATTCACTATGATGAAAAAAACAATGAATGGAACCTGCTCCAAGTTA
AAAATAGAGATAATACCGAAAACTCATCGAGTAGTAAGATTAGAGATAATACAACAATAAAAAAA
TGGTTTAGAACTTACTCACAGCGTGATGCTACTAATTGGGACAATTTTCCAGATGAAGTATCATCTA
AGAATTTAAATGAAGAAGACTTCAGAGCTTTTGTTAAAAATTATTTGGCAAAAATAATATAATTCG
GCTGCAGGGGCGGCCTCGTGATACGCCTATTTTTATAGGTTAATGTCATGATAATAATGGTTTCTTA
GACGTCAGGTGGCACTTTTCGGGGAAATGTGCGCGGAACCCCTATTTGTTTATTTTTCTAAATACAT
TCAAATATGTATCCGCTCATGAGACAATAACCCTGATAAATGCTTCAATAATATTGAAAAAGGAAG
AGTATGAGTATTCAACATTTCCGTGTCGCCCTTATTCCCTTTTTTGCGGCATTTTGCCTTCCTGTTTTT
GCTCACCCAGAAACGCTGGTGAAAGTAAAAGATGCTGAAGATCAGTTGGGTGCACGAGTGGGTTA
CATCGAACTGGATCTCAACAGCGGTAAGATCCTTGAGAGTTTTCGCCCCGAAGAACGTTTTCCAAT
GATGAGCACTTTTAAAGTTCTGCTATGTGGCGCGGTATTATCCCGTATTGACGCCGGGCAAGAGCA
ACTCGGTCGCCGCATACACTATTCTCAGAATGACTTGGTTGAATACTCACCAGTCCAGNAAAGCAT
CTTACGGATGGCTTGACCGTAAGAAAATTATGCAGGGCTGCCATAACCTTGAATGAACCCTGGCGC
CAANTTACTTTGAAAACGATCGGGGGANNAAAGGAACTACCCCTTTTTTGCCANNNGGGGGAAAT
ATTAATCCCCCCTTNNTTTTGNGAANNNNGTAAAAAACCTCCCNNAACAAAGAGGGGCCCCCAGTG
CTGTA

Gene ID: Glyma.12G165301.Wm82.a4.v1 ➔ 15 exons

CircRNA ID: Gm12:33422727-33423151 ➔ exons 10 - 12

Exon 10

ATCCTGCATGCTACCAAAACAAACAAGAATGCCTTCAAGACTCTGATTGCGGCA[R:GAGTACAGTGGCGTCCAAGTGGA]ATTGGCCCCTAATT
TCGAGATGGGTGTCTCTAACAAAACTCCTGAATTTCTCAAGATGAATCCTATTGGAAAG

Exon 11

GTTCC[F:TGTTTTGGAAACGCCCGATGGTCC]AGTCTTTGAGAGCAATGCAATCGCTCGTTACG

Exon 12

TTGCTCGACTAAAGGGTGACAACGCTTTGTTCTCATCTTCTGCCATTGATAAT

CircRNA junction:

[F:TGTTTTGGAAACGCCCGATGGTCC]AGTCTTTGAGAGCAATGCAATCGCTCGTTACGTTGCTCGACTAAAGGGTGACAACGCTTTGTTCTCAT
CTTCTGCCATTGATAATATCCTGCATGCTACCAAAACAAACAAGAATGCCTTCAAGACTCTGATTGCGGCA[R:GAGTACAGTGGCGTCCAAGT
GGA]

Gel electrophoresis and Sanger Sequencing:

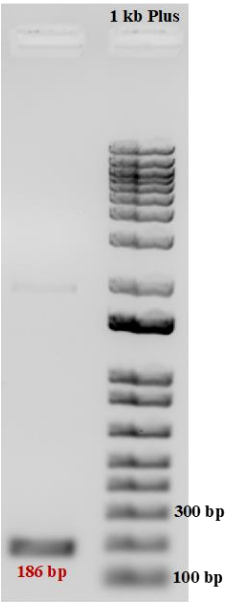

CCCGAAATGTAGGAGATCTTCTAGAAAGAT[F:TGTTTTGGAAACGCCCGATGGTCC]AGTCTTTGAG
AGCAATGCAATCGCTCGTTACGTTGCTCGACTAAAGGGTGACAACGCTTTGTTCTCATCTTCTGCCA
TTGATAATATCCTGCATGCTACCAAAACAAACAAGAATGCCTTCAAGACTCTGATTGCGGCA[R:GA
GTACAGTGGCGTCCAAGTGGA]ATCTTGCTGAAAAACTCGAGCCATCCGGAAGATCTGGCGGCCGC
TCTCCCTATAGTGAGTCGTATTACGCCGGATGGATATGGTGTTCAGGCACAAGTGTTAAAGCAGTT
GATTTTATTCACTATGATGAAAAAAACAATGAATGGAACCTGCTCCAAGTTAAAAATAGAGATAAT
ACCGAAAACTCATCGAGTAGTAAGATTAGAGATAATACAACAATAAAAAAATGGTTTAGAACTTA
CTCACAGCGTGATGCTACTAATTGGGACAATTTTCCAGATGAAGTATCATCTAAGAATTTAAATGA
AGAAGACTTCAGAGCTTTTGTTAAAAATTATTTGGCAAAAATAATATAATTCGGCTGCAGGGGCGG
CCTCGTGATACGCCTATTTTTATAGGTTAATGTCATGATAATAATGGTTTCTTAGACGTCAGGTGGC
ACTTTTCGGGGAAATGTGCGCGGAACCCCTATTTGTTTATTTTTCTAAATACATTCAAATATGTATC
CGCTCATGAGACAATAACCCTGATAAATGCTTCAATAATATTGAAAAAGGAAGAGTATGAGTATTC
AACATTTCCGTGTCGCCCTTATTCCCTTTTTTGCGGCATTTTGCCTTCCTGTTTTTGCTCACCCAGAA
ACGCTGGTGAAAGTAAAAGATGCTGAAGATCAGTTGGGTGCACGAGTGGGTTACATCGAACTGGA
TCTCAACAGCGGTAAGATCCTTGAGAGTTTTCGCCCCGAAGAACGTTTTCCAATGATGAGCACTTTT
AAAGTTCTGCTATGTGGCGCGGTATTATCCCGTATTGACGCCGGGCAAGAGCAACTCGGTCGCCGC
ATACACTATTCTCAGAATGACTTGGTTGAGTACTCCCCAGTCANNGAAAAGCATCTTACGGATGGC
TGACCGTAAGAGAATTATGCAGTGCTGCCNTACCATGAGGGAAAACCTGGGGCCAATTACTTNGAN
ACGATCGGAGGACCAAGGAGCTAACNCTTTTTNGCCCACNGGGGGAANTGGAATCGCCTTGATCTT
TGGAACCGAACTAAAAAACCCTCCAACCCCCNCGGGACCCCAGCCCTAAGGGGAACCTTGGCAAA
TTTAGGGGGAAATTTTCTCCTTCCC

Gene ID: Glyma.19G250600.Wm82.a4.v1 ➔ 30 exons

CircRNA ID: Gm19:50184151-50188011 ➔ exons 17 - 23

Exon 17:

CAACTGTTGCAACATTGACAGATCTTGGTTTCTTATGGTTTAGAGAATTCTATTTAGAGTCTTCACGAGTCATTCAG

Exon 18:

TTTCCAATTGAATGCTCCCTTCCTTGGATGTTGGTGGATTGTGTACTTGAGTCACCGAATTCTGGTCTTCTCGAGAGTGTTCTAATGCCATTTGA[
R:CATCTATAATGATTCAGCTCAGCAAGCCTT]GGTGTTGCTGAAGCAACGGTTTTTATACGATGAAATTGAGGCTGAG

Exon 23:

ATTTGGTCTGAGATGCATAGTGATTTTTT[F:GCCAAATTTCATTCTCTGCAATACTACTCAACG]TTTTATCAGATCATCAAGAACGGTTCCTGTT
CAAAAGCCATCTGTACCCTCTTCTAAGCCTAGTTTCTATTGTGGTACTCAA

CircRNA junction:

[F:GCCAAATTTCATTCTCTGCAATACTACTCAACG]TTTTATCAGATCATCAAGAACGGTTCCTGTTCAAAAGCCATCTGTACCCTCTTCTAAGC
CTAGTTTCTATTGTGGTACTCAACAACTGTTGCAACATTGACAGATCTTGGTTTCTTATGGTTTAGAGAATTCTATTTAGAGTCTTCACGAGTCA
TTCAGTTTCCAATTGAATGCTCCCTTCCTTGGATGTTGGTGGATTGTGTACTTGAGTCACCGAATTCTGGTCTTCTCGAGAGTGTTCTAATGCCA
TTTGA[R:CATCTATAATGATTCAGCTCAGCAAGCCTT]

Gel electrophoresis and Sanger Sequencing:

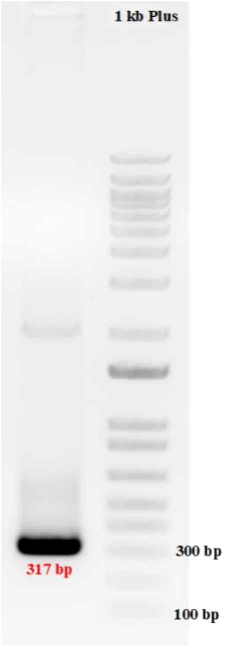

CCCGAACTCGGAATGGCTCGAGTTTTNAGCAAGAT[F:GCCAAATTTCATTCTCTGCAATACTACTCA
ACG]TCTTATCAGATCATCAAGAACGGTTCCTGTTCAAAAGCCATCTGTACCCTCTTCTAAGCCTAG
TTTCTATTGTGGTACTCAACAACTGTTGCAACATTGACAGATCTTGGTTTCTTATGGTTTAGAGAAT
TCTATTTAGAGTCTTCACGAGTCATTCAGTTTCCAATTGAATGCTCCCTTCCTTGGATGTTGGTGGA
TTGTGTACTTGAGTCACCGAATTCTGGTCTTCTCGAGAGTGTTCTAATGCCATTTGA[R:CATCTATA
ATGATTCAGCTCAGCAAGCCTT]ATCTTTCTAGAAGATCTCCTACAATATTCTCAGCTGCCATGGAA
AATCGATGTTCTTCTTTTATTCTCTCAAGATTTTCAGGCTGTATATTAAAACTTATATTAAGAACTAT
GCTAACCACCTCATCAGGAACCGTTGTAGGTGGCGTGGGTTTTCTTGGCAATCGACTCTCATGAAA
ACTACGAGCTAAATATTCAATATGTTCCTCTTGACCAACTTTATTCTGCATTTTTTTTGAACGAGGT
TTAGAGCAAGCTTCAGGAAACTGAGACAGGAATTTTATTAAAAATTTAAATTTTGAAGAAAGTTCA
GGGTTAATAGCATCCATTTTTTTGCTTTGCAAGTTCCTCAGCATTCTTAACAAAAGACGTCTCTTTTG
ACATGTTTAAAGTTTAAACCTCCTGTGTGAAATTATTATCCGCTCATAATTCCACACATTATACGAG
CCGGAAGCATAAAGTGTAAAGCCTGGGGTGCCTAATGAGTGAGCTAACTCACATTAATTGCGTTGC
GCTCACTGCCAATTGCTTTCCAGTCGGGAAACCTGTCGTGCCAGCTGCATTAATGAATCGGCCAAC
CGCGGGGAGAGGCGGTTTGCGTATTGGGCGGCCTTCCGCTTCCTCCATCACCGA

Gene ID: Glyma.05G181000.Wm82.a4.v1 ➔ 10 exons

CircRNA ID: Gm05:36914887-36915270 ➔ exons 2 and 3

Exon 2:

CTTTTAGCTTGTGACAAATCGCTTGGCGCAGTTCCATGGTCCCGG[R:CATTGGGCGTGTACCTCGTGTAACC]TTCACGAATTGCATTAATCCCA
GC

Exon 3:

CTCGGCTATGGGAGCGGGCGTGTCGAAATCGGGCTCGCCGGCGGCGAGGCGGATGACGGGAACGCCGGCTTGGACGAGAGCGGTGGCGTGGT
CGCTGATGGCGACGGTTT[F:TGGAAGGCTTGACGGCATTGACAC]GAGGACTGAGCGAAAGGTCAACGTCGAAGTCAGAGTGCGAAGAAGCCT
TAACCGCAATTGCGTTGGGTTGTTTTGCCGCACGTGTT

CircRNA junction:

[F:TGGAAGGCTTGACGGCATTGACAC]GAGGACTGAGCGAAAGGTCAACGTCGAAGTCAGAGTGCGAAGAAGCCTTAACCGCAATTGCGTTGG
GTTGTTTTGCCGCACGTGTTCTTTTAGCTTGTGACAAATCGCTTGGCGCAGTTCCATGGTCCCGG[R:CATTGGGCGTGTACCTCGTGTAACC]

Gel electrophoresis and Sanger Sequencing:

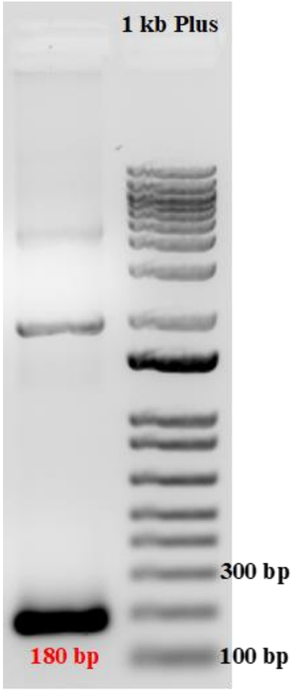

GTTTAAATANGGGGCACCCTCCTNNNNCTCTCATTTTTNNNAAANAACCCCNNGGGAGGGGG
GGGGGGGGAGGGGTTCCGACTTTTTCCCCCCCCNNNCTTTTTTATTTGTNCGGANATAGAGAA
ATTTTCCCCATTAATTTTGGCAAAGTTTTCCCTTTTNCGGATTGGGGTTCCCGTCTNNTNNTTG
GTNGGGTTCATCAGTCGGTTCCCAAGATCAGGCGAGTTACAGATCCCCCAGTTGGCAAAAAG
CGGTAGTTCCTTGGTCCTCCGATCGTGTCGAAGTAAGTGGCCGCAGNTTATCACTCAGGTTAG
GCAGCACTGCATAATTTTTTTACTGTCATGCCATCCGTAAGATGCTTTCTGTGACTGGTGAGTA
CTCAACCAAGTCATTCTGAGAATAGTGTATGCGGCGACCGAGTTGCTCTTGCCCGGCGTCAAT
ACGGGATAATACCGCGCCACATAGCAGAACTTTAAAAGTGCTCATCATTGGAAAACGTTCTT
CGGGGCGAAAACTCTCAAGGATCTTACCGCTGTTGAGATCCAGTTCGATGTAACCCACTCGTG
CACCCAACTGATCTTCAGCATCTTTTACTTTCACCAGCGTTTCTGGGTGAGCAAAAACAGGAA
GGCAAAATGCCGCAAAAAAGGGAATAAGGGCGACACGGAAATGTTGAATACTCATACTCTTC
CTTTTTCAATATTATTGAAGCATTTATCAGGGTTATTGTCTCATGAGCGGATACATATTTGAAT
GTATTTAGAAAAATAAACAAATAGGGGTTCCGCGCACATTTCCCCGAAAAGTGCCACCTGAC
GTCTAAGAAACCATTATTATCATGACATTAACCTATAAAAATAGGCGTATCACGAGGCCGCC
CCTGCAGCCGAATTATATTATTTTTGCCAAATAATTTTTAACAAAAGCTCTGAAGTCTTCTTCA
TTTAAATTCTTAGATGATACTTCATCTGGAAAATTGTCCCAATTAGTAGCATCACGCTGTGAG
TAAGTTCTAAACCATTTTTTTATTGTTGTATTATCTCTAATCTTACTACTCGATGAGTTTTCGGT
ATTATCTCTATTTTTAACTTGGAGCAGGTTCCATTCATTGTTTTTTTCATCATAGTGAATAAAA
TCAACTGCTTTAACACTTGTGCCTGAACACCATATCCATCCGGCGTAATACGACTCACTATAG
GGAGAGCGGCCGCCAGATCTTCCGGATGGCTCGAGTTTTTCAGCAAGAT[F:TGGAAGGCTTGA
CGGCATTGACAC]GAGGACTGAGCGAAAGGTCAACGTCGAAGTCAGAGTGCGAAGAAGCCTT
AACCGCAATTGCGTTGGGTTGTTTTGCCGCACGTGTTCTTTTAGCTTGTGACAAATCGCTTGGC
GCAGTTCCATGGTCCCGG[R:CATTGGGCGTGTACCTCGTGTAACC]ATCTTNCTAGAAGATCTC
CTACATTTCGGG

Gene ID: Glyma.09G072200.Wm82.a4.v1➔ 14 exons

CircRNA ID: Gm09:7441707-7442379➔ exons 6 - 8

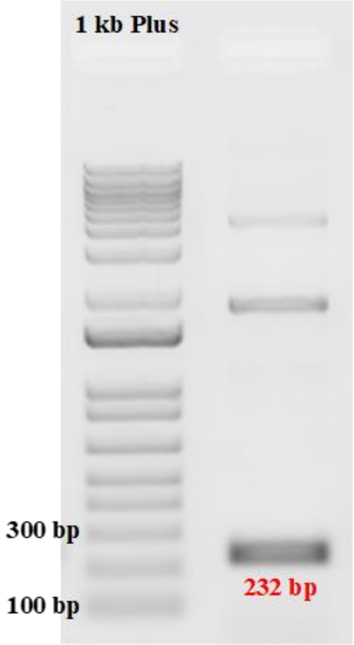

Gene ID: Glyma.09G002100.Wm82.a4.v1➔ 22 exons

CircRNA ID: Gm09:159108-159502➔ exons 11 and 12

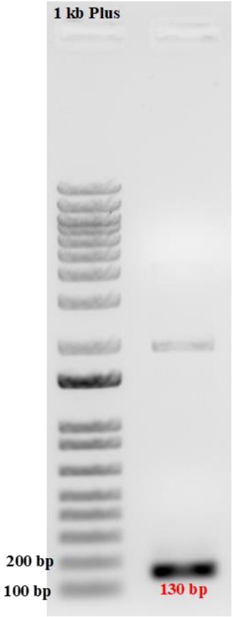

Gene ID: Glyma.13G328100.Wm82.a4.v1➔ 18 exons

CircRNA ID: Gm13:41646492-41647810➔ exons 8 - 12

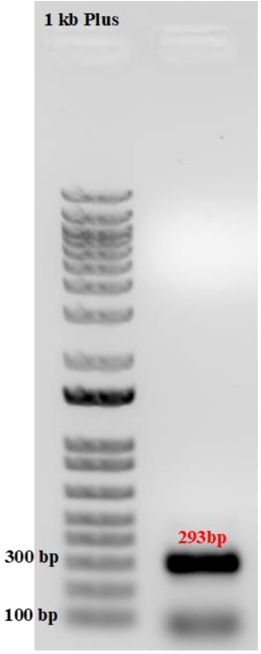

Gene ID: Glyma.20G174900.Wm82.a4.v1➔ 22 exons

CircRNA ID: Gm20:41203647-41204058➔ exons 12 and 13

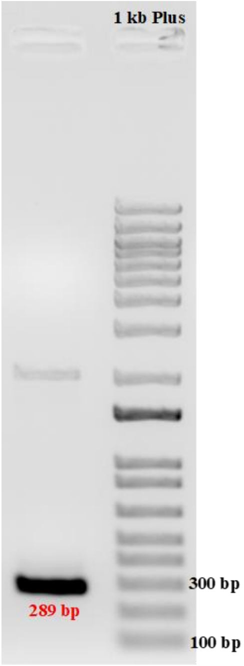

Gene ID: Glyma.13G174600.Wm82.a4.v1➔ 47 exons

CircRNA ID: Gm13:28305198-28306795➔ exons 19 - 23

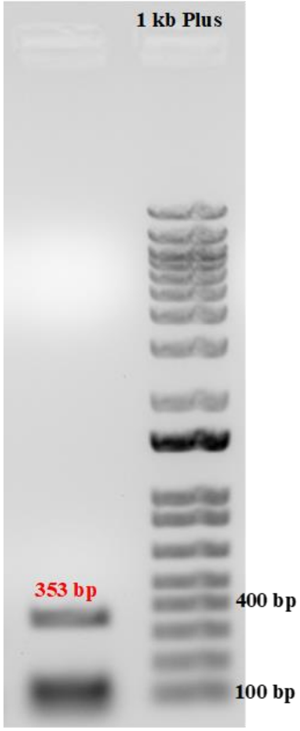

Gene ID: Glyma.11G165900.Wm82.a4.v1➔ 22 exons

CircRNA ID: Gm11:21290647-21291065➔ exons 12 - 13

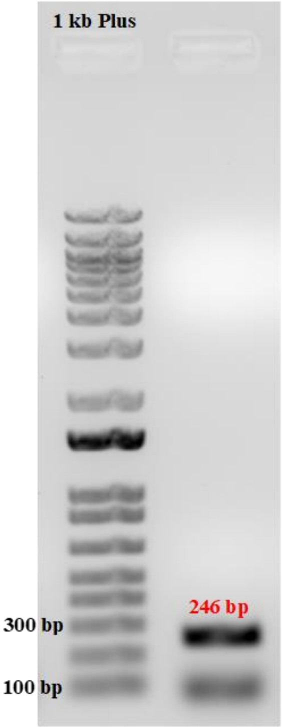

Gene ID: Glyma.04G192300.Wm82.a4.v1➔ 44 exons

CircRNA ID: Gm04:45203596-45204445➔ exons 4 – 8

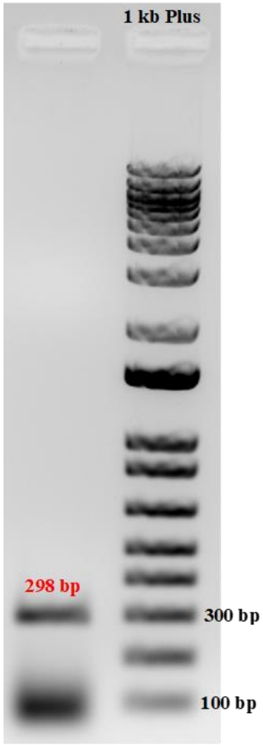

Gene ID: Glyma.06G268800.Wm82.a4.v1➔ 16 exons

CircRNA ID: Gm06:45365226-45365827➔ exons 9 - 10

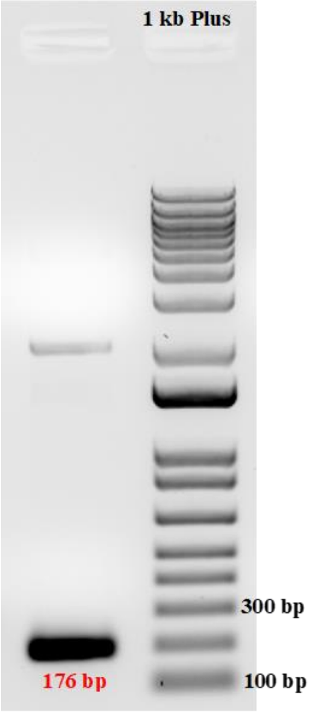

Gene ID: Glyma.01G188900.Wm82.a4.v1➔ 5 exons

CircRNA ID: Gm01:53520281-53520651➔ exons 3 - 4

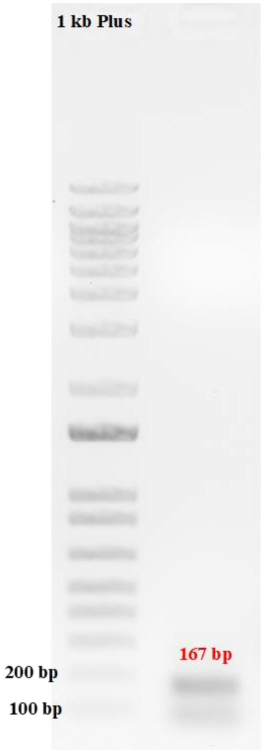

Gene ID: Glyma.02G164700.Wm82.a4.v1➔ 18 exons

CircRNA ID: Gm02:25770068-25770721➔ exons 15 - 17

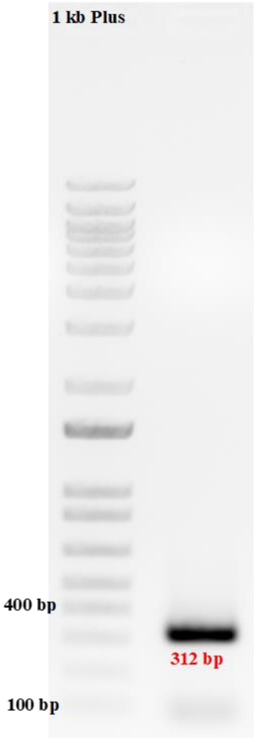

Gene ID: Glyma.01G086201.Wm82.a4.v1➔ 15 exons

CircRNA ID: Gm01:25633923-25634953➔ exons 8 - 9

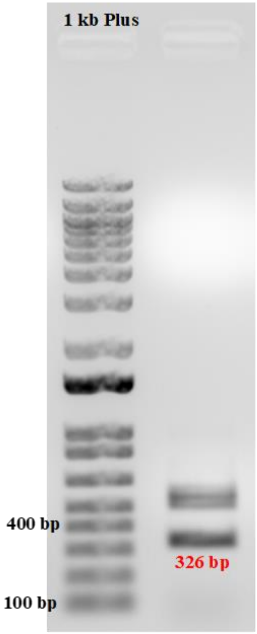

Gene ID: Glyma.20G230200.Wm82.a4.v1➔ 9 exons

CircRNA ID: Gm20:46371201-46371776➔ exons 2 - 4

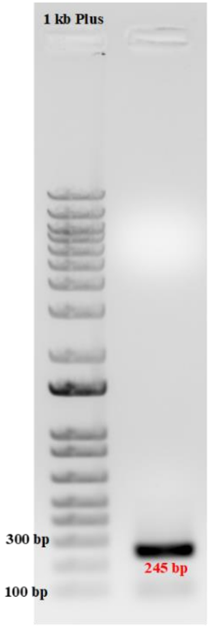

Gene ID: Glyma.15G130100.Wm82.a4.v1➔ 5 exons

CircRNA ID: Gm15:10387255-10388352➔ exons 2 - 3

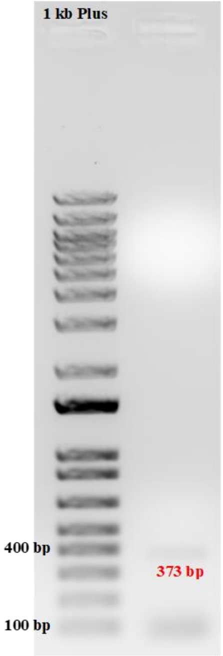

Validation of circRNAs Using Divergent Primers and PCR: Full-Length Agarose Gel Results

We only assigned numbers to the bands used in our results report and publication. Full detail for every validated circRNA can be found above.

Gel_1

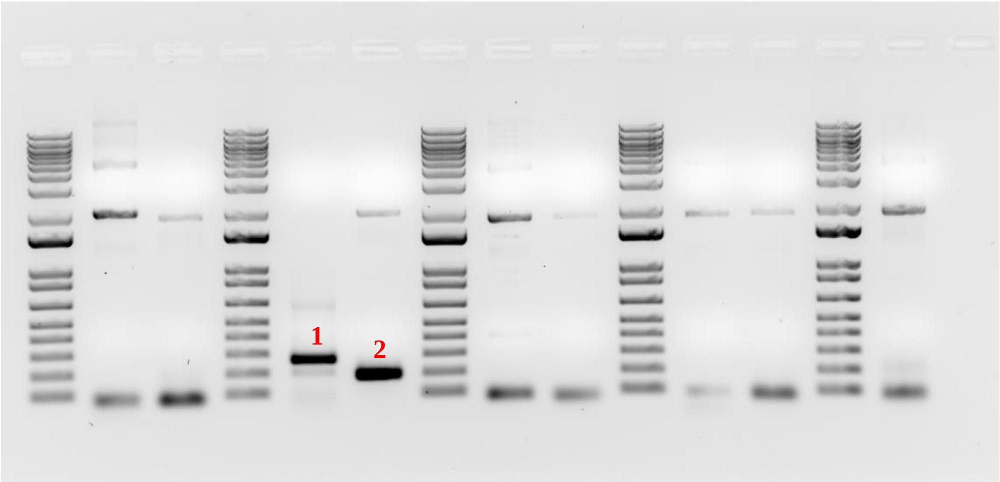

CircRNA IDs for Gel_1:

1. Gm05:92631-94898 (269bp)

2. Gm06:45365226-45365827 (176bp)

Gel_2

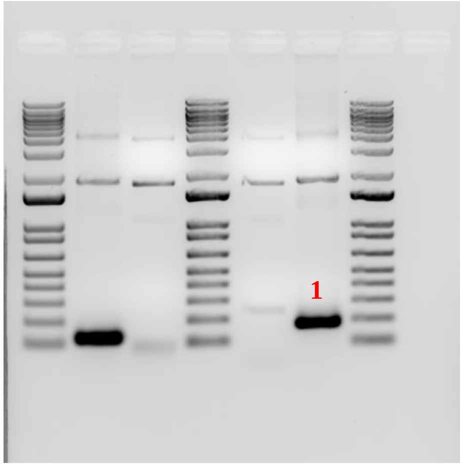

CircRNA IDs for Gel_2:

1. Gm05:36914887-36915270 (180 bp)

Gel_3

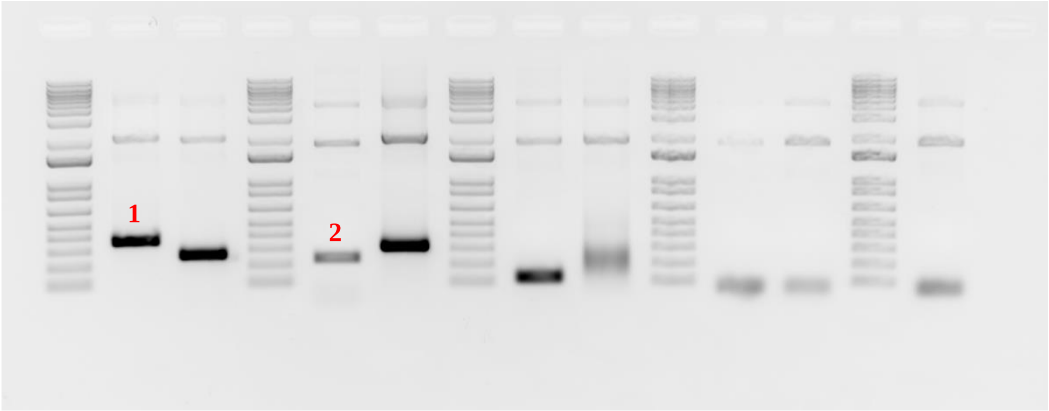

CircRNA IDs for Gel_3:

1. Gm13:25614859-25616046 (367bp)

2. Gm09:7441707-7442379 (232bp)

Gel_4

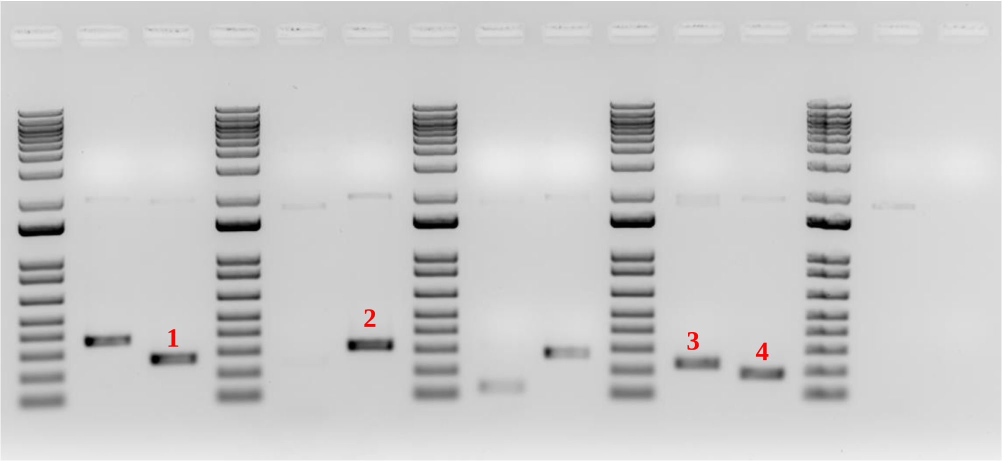

CircRNA IDs for Gel_4:

1. Gm04:45203808-45204445 (263bp)

2. Gm11:3832979-3834119 (318bp)

3. Gm06:33087-34718 (226bp)

4. Gm12:33422727-33423151 (186bp)

Gel_5

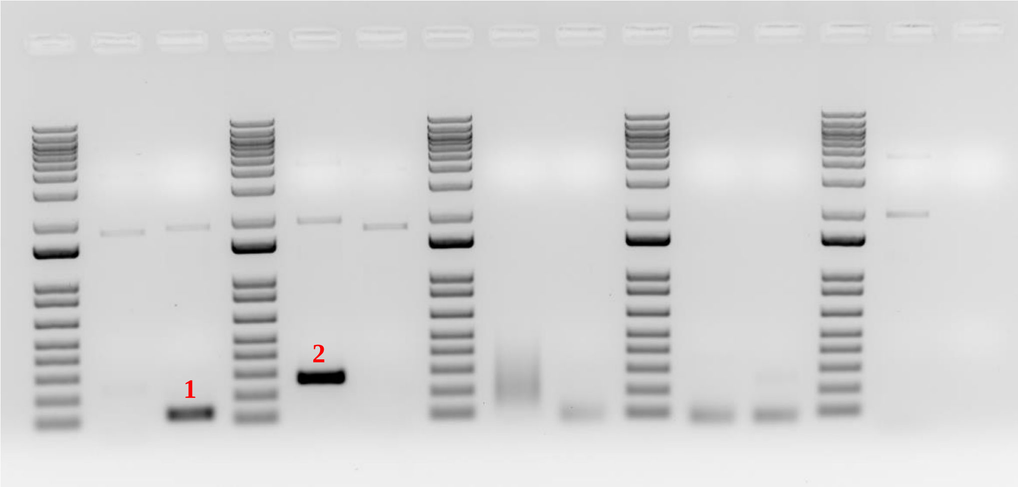

CircRNA IDs for Gel_5:

1. Gm04:60681-61366 (118bp)

2. Gm17:41684169-41684774 (272bp)

Gel_6

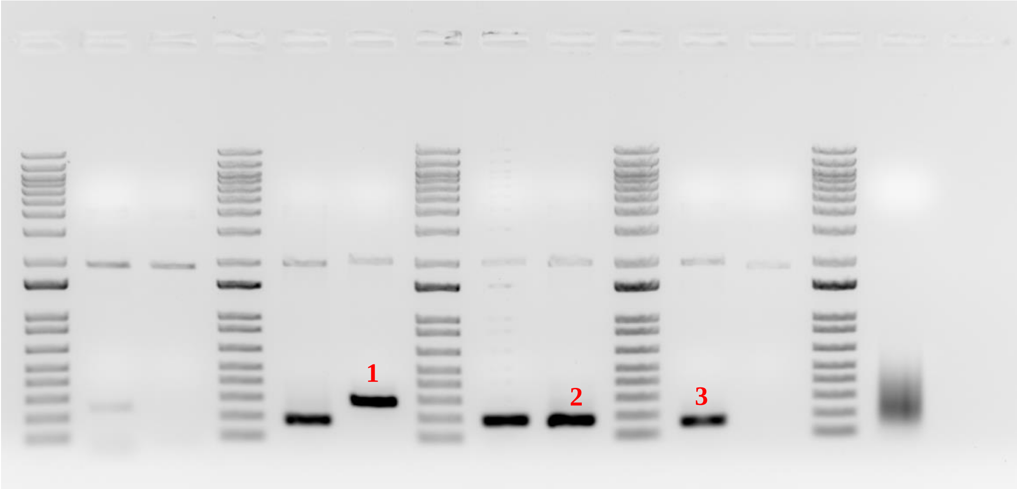

CircRNA IDs for Gel_6:

1. Gm20:41203647-41204058 (289bp)

2. Gm08:2820041-2820979 (175bp)

3. Gm09:159108-159502 (130bp)

Gel_7

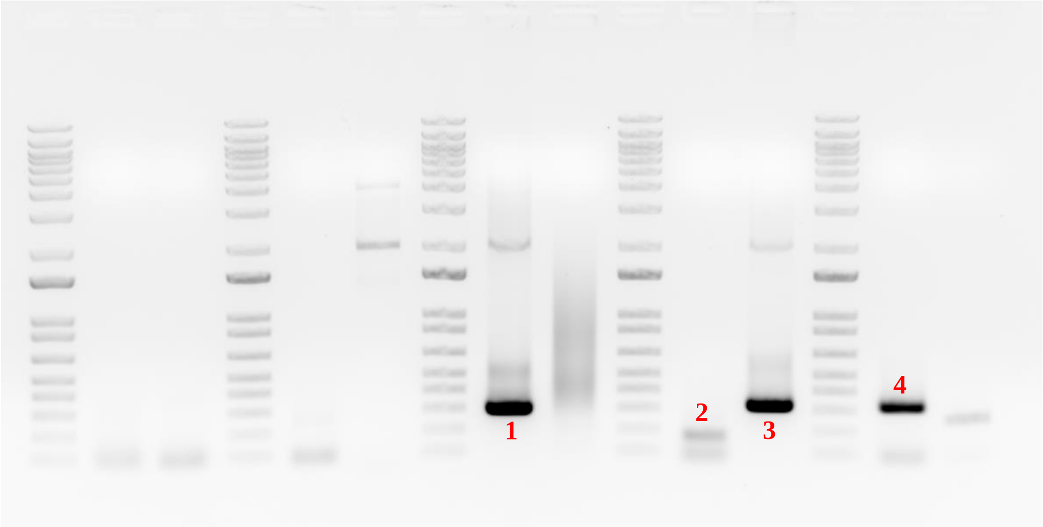

CircRNA IDs for Gel_7:

1. Gm05:9376287-9377127 (292bp)

2. Gm01:53520281-53520651 (167bp)

3. Gm19:50184151-50188011 (317bp)

4. Gm02:25770068-25770721 (312bp)

Gel_8

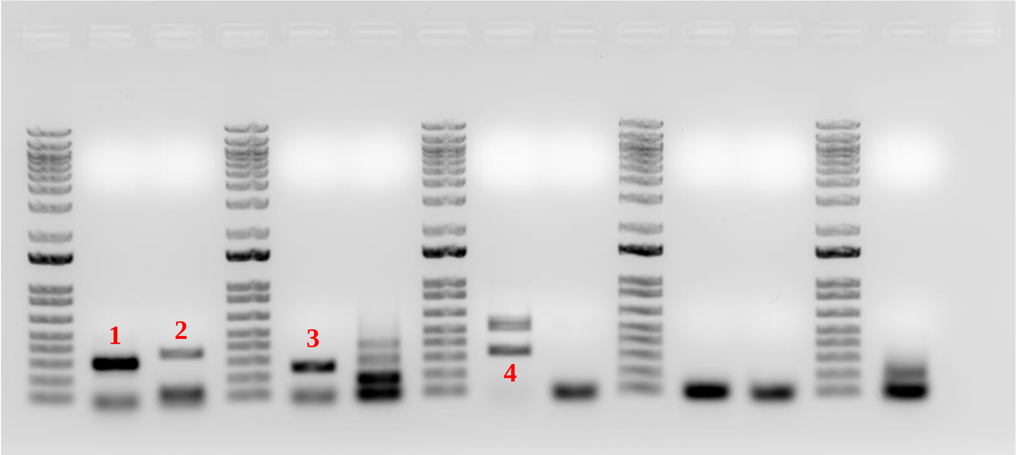

CircRNA IDs for Gel_8:

1. Gm13:41646492-41647810 (293bp)

2. Gm13:28305198-28306795 (353bp)

3. Gm11:21290647-21291065 (246bp)

4. Gm01:25633923-25634953 (326bp)

Gel_9

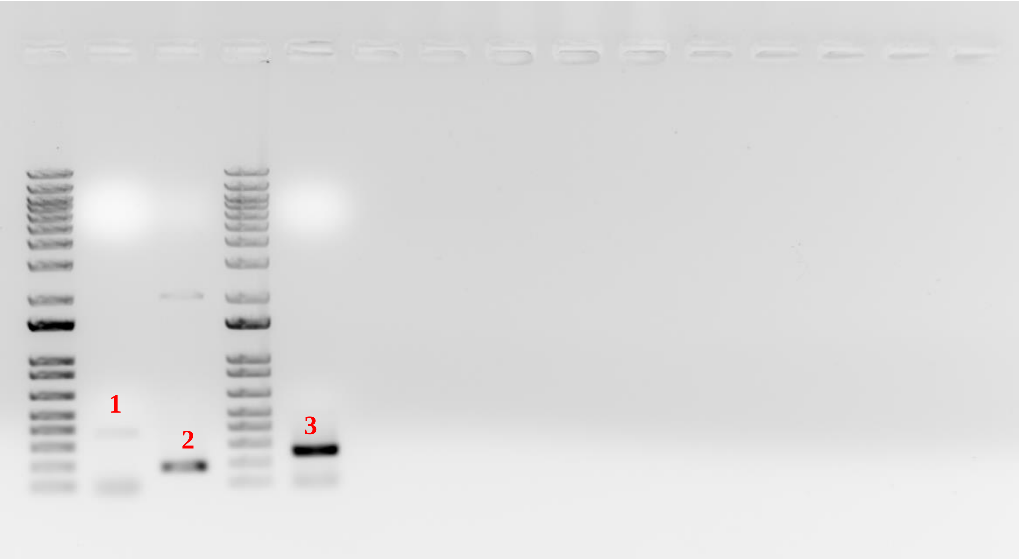

CircRNA IDs for Gel_9:

1. Gm15:10387255-10388352 (373bp)

2. Gm19:46460019-46460732 (183bp)

3. Gm20:46371201-46371776 (245bp)

Gel_10

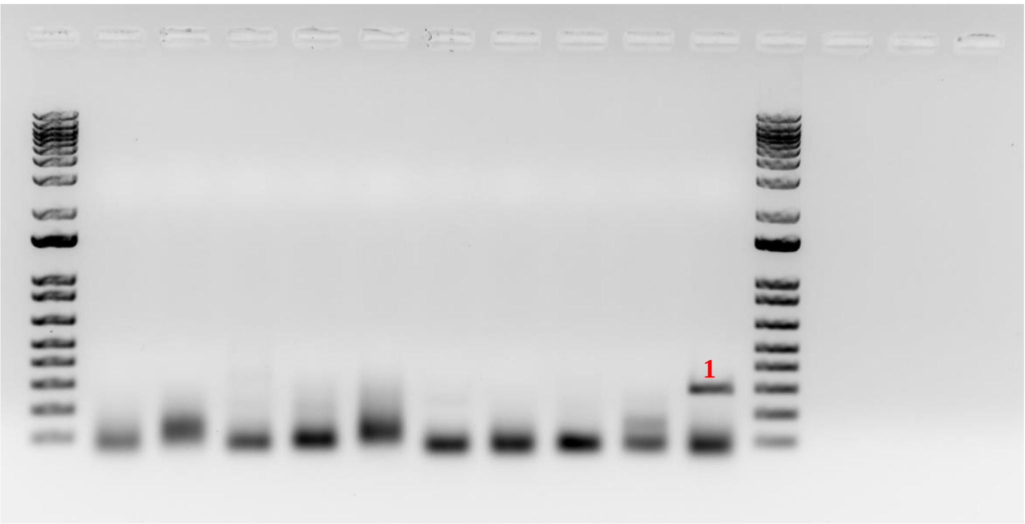

CircRNA IDs for Gel_10:

1. Gm04:45203596-45204445 (298bp)
